# MAPtools: command-line tools for mapping-by-sequencing and QTL-Seq analysis and visualization

**DOI:** 10.1186/s13007-024-01222-2

**Published:** 2024-07-17

**Authors:** César Martínez-Guardiola, Ricardo Parreño, Héctor Candela

**Affiliations:** Instituto de Bioingeniería, Universidad Miguel Hernández de Elche, Campus de Elche, Elche, 03202 Spain

## Abstract

**Background:**

Classical mutagenesis is a powerful tool that has allowed researchers to elucidate the molecular and genetic basis of a plethora of processes in many model species. The integration of these methods with modern massively parallel sequencing techniques, initially in model species but currently also in many crop species, is accelerating the identification of genes underlying a wide range of traits of agronomic interest.

**Results:**

We have developed MAPtools, an open-source Python3 application designed specifically for the analysis of genomic data from bulked segregant analysis experiments, including mapping-by-sequencing (MBS) and quantitative trait locus sequencing (QTL-seq) experiments. We have extensively tested MAPtools using datasets published in recent literature.

**Conclusions:**

MAPtools gives users the flexibility to customize their bioinformatics pipeline with various commands for calculating allele count-based statistics, generating plots to pinpoint candidate regions, and annotating the effects of SNP and indel mutations. While extensively tested with plants, the program is versatile and applicable to any species for which a mapping population can be generated and a sequenced genome is available.

**Availability and implementation:**

MAPtools is available under GPL v3.0 license and documented as a Python3 package at https://github.com/hcandela/MAPtools.

**Supplementary Information:**

The online version contains supplementary material available at 10.1186/s13007-024-01222-2.

## Introduction

Mapping-by-sequencing (MBS) is a powerful technique that combines high-throughput sequencing technologies and bulked segregant analysis to rapidly map mutations identified in mutagenesis screens. This approach was initially developed for model organism research [1] and holds great promise for enhancing our understanding of complex biological systems, as extensively reviewed by us and other authors [2–5]. Another related technique, called quantitative trait loci sequencing (QTL-seq), has been proposed for the rapid mapping of QTLs in species with a sequenced genome and is accelerating the identification of genomic regions associated with traits of agricultural interest in many crops, often in combination with other experimental approaches, such as classical linkage and QTL mapping or the identification of differentially expressed genes by RNA-seq [6–8].

In this article, we introduce MAPtools, a collection of command-line utilities designed to analyze data from MBS and QTL-seq experiments. A growing number of software tools and analysis pipelines for MBS and QTL-seq data have been released in recent years [9–13], but many of them are limited in terms of the analyses they perform or the species they focus on. Other programs, such as CandiSNP, SIMPLE and Easymap, have prioritized ease of use by non-expert users [14–16]. MAPtools, by contrast, has been designed to be a versatile tool that can receive input data from a stream, giving the users maximum flexibility in their choice of read mappers and variant callers. The versatility of MAPtools is illustrated by its ability to analyze data from different segregating populations and crossing schemes. Using simulated reads, previous authors have extensively studied how the identification of mutated genes depends on the sequencing depth and bulk size, two important factors that users must also consider when designing MBS experiments [17, 18]. The syntax of MAPtools is straightforward and similar to that of other highly popular and widely used programs such as SAMtools or BCFtools, which should make it user-friendly for researchers who are already familiar with these programs. For this reason, the learning curve for the program is not expected to pose a significant barrier to new users.

## Implementation

### The MAPtools application

MAPtools is a Python3 v. 3.8-based, standalone application that is distributed under the GPL v3.0 license and freely available to users. Its dependencies are limited to several packages commonly used in scientific computing, including docopt (v. 0.6.2), NumPy (v. 1.24.2), SciPy (v. 1.10.1), pandas (v. 2.0.0), biopython (v. 1.81) and matplotlib (v. 3.7.1), which can be easily installed with pip (v. 20.0.2). The program has been tested with the latest versions of the libraries available at the time of submission. Its distribution through a GitHub repository (https://github.com/hcandela/MAPtools) will facilitate the long-term maintenance of the code. We developed and tested the program on a desktop computer equipped with two Intel(R) Xeon(R) CPU E5-2620 v4 @ 2.10 GHz (16 cores, 32 threads) processors and 125 Gi RAM, but the program has also been successfully tested on computers with less memory or fewer cores.

**Fig. 1:**
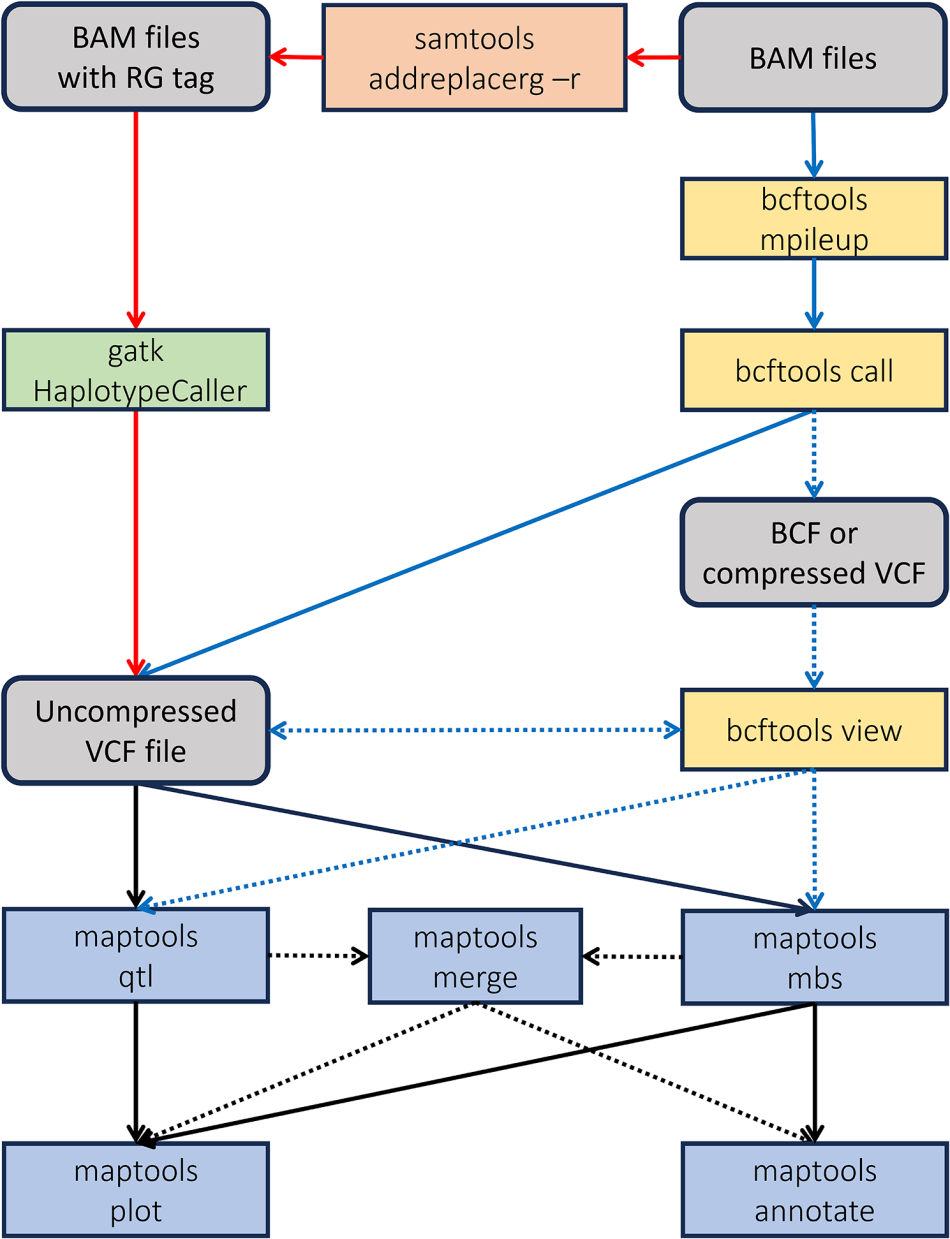
MBS and QTL-seq workflows using MAPtools. MAPtools requires data in uncompressed VCF format, which can be read from disk or from a stream. Data in this format can be produced either by BCFtools or by GATK, and serves as input for MAPtools’ qtl and mbs commands. Data in other formats can be converted to uncompressed VCF by BCFtools’ view command. This command can also be used to apply additional quality or sequencing depth filters to the uncompressed VCF data. The output of qtl and mbs can serve as input for other MAPtool’s commands, like merge, plot and annotate

The ability to integrate MAPtools in workflows with other tools gives researchers maximum control over their analysis pipelines (Fig. 1). For example, users may choose to filter read pairs based on mapping quality or sequencing depth prior to using MAPtools, or they may want to include extra steps to mark or remove duplicate read pairs that, if present, might bias the real allele frequencies. A typical QTL-seq or MBS analysis pipeline involves aligning reads from distinct samples to a well-annotated reference genome using software tools such as BWA [19] or Bowtie2 [20]. The resulting files in sequence alignment/map format (SAM) [21] are then converted to compressed binary format (BAM) and processed using variant calling software, such as BCFtools [22, 23] or GATK [24, 25]. MAPtools can directly read input data in uncompressed Variant Call Format (VCF), provided that it includes allelic depth (AD) fields for each sample in separate columns. VCF data with AD fields is produced by BCFtools’ mpileup command when it is run with the ––annotate option, and also by GATK’s HaplotypeCaller if the BAM files contain different RG fields for each sample. We have tested the program with input files produced by BCFtools’ call command, which can also output data to a stream, and by GATK’s HaplotypeCaller command, but MAPtools should also work with any other software that outputs data in VCF format. Although the current version of MAPtools cannot directly read input data from compressed or uncompressed BCF files or from compressed VCF files, this can be easily achieved by including a conversion step in the workflow using BCFtools’ view command. The amount of RAM required by MAPtools is quite low, particularly when the input VCF file contains only the variant sites.

The initial release of MAPtools (v. 1.0) includes six commands that support the analysis of MBS and QTL-seq data. Specifically, two commands, namely mbs and qtl, enable the analysis of data from MBS or QTL-seq experiments and calculate different statistics depending on the type of experiment and the available input dataset. The plot command plots the results and creates publication-quality figures with their captions. Additionally, the merge command can integrate the allele counts from all markers within a window, allowing the output to refer to haplotypes rather than individual markers. The annotate command allows users to assess the effect of all candidate mutations within a user-selected interval. Lastly, the citation command provides information on the version of the program in use. The mbs, qtl and merge commands produce output in a VCF-like format, which can be read by the merge and plot commands. This output consists of a header similar to that of the VCF files, containing information about the program, the options used, as well as the headings and a description of the contents of each column. The header cumulatively records each step performed, which should help to improve its reproducibility.

## MAPtools commands

### Analysis of MBS data

The mbs command processes the VCF input to tabulate the allele counts and calculate different parameters that facilitate identifying a mutation of interest through MBS. The user must designate each available sample using option -d (--data) as one of the following: R (the required bulk of phenotypically recessive individuals from the mapping population), D (an optional bulk of phenotypically dominant individuals from the mapping population), Pr (the phenotypically recessive parent of the mapping population) and Pd (the phenotypically dominant parent of the mapping population). Depending on the available samples, the calculated parameters might include the SNP-index (defined as the frequency of alleles inherited from the phenotypically recessive progenitor), the ∆(SNP-index) (defined as the difference of the SNP-indices in the D and R bulks), the exact probabilities and *p*-values of Fisher’s exact tests, the Euclidean distances calculated for individual markers (ED_m_) and the G statistic, all of which have previously been used in BSA-seq experiments [26]. When the reference genome sequence matches that of one parent of the mapping population, or if one or both parents of the mapping population have been resequenced, the mbs command uses this information to classify the alleles based on their parental origin: The program can handle the following experimental situations:

(i) *With genomic DNA from the R bulk*. The user can specify if the alleles in the reference sequence match those of the dominant or the recessive parent, enabling the calculation of allele frequencies (AFs, also known as SNP-indices) for the alleles inherited from the phenotypically recessive parent. If such information is unavailable, the program will instead calculate the AF for the most abundant allele, regardless of its parental origin, yielding values equal to or greater than 0.5.
(ii) *With genomic DNA from the R and D bulks*. If the reference genome sequence matches the dominant or the recessive parent, the program will additionally report other parameters useful for comparing the allele counts in the two bulks. Like in the previous case, the command reports the AF for the most abundant allele in the recessive bulk when the parental origin of alleles is unknown.
(iii) *With genomic DNA from the R bulk plus one parent (Pd or Pr).* In this scenario, the sequence of the parent allows determining the parental origin of the alleles, enabling the calculation of the AF for alleles inherited from the recessive parent. Resequencing of at least one of the parents is particularly recommended when the reference genome does not match any of the parents of the mapping population.
(iv) *With genomic DNA from the R and D bulks plus one parent (Pd or Pr)*. Similar to case (ii), this setup enables the calculation of the AF for alleles from the recessive parent, as well as other parameters that require two bulks from the mapping population.
(v) *With genomic DNA from the R bulk plus the two parents (Pd and Pr)*. This case differs from case (iii) in that the resequencing of the two parents is supplied as input. In this situation, the program will focus on the polymorphisms detected between the two parents and proceed as usual.
(vi) *With genomic DNA from the R and D bulks plus the two parents (Pd and Pr)*. Similar to case (v), this case differs from case (iv) in that the resequencing of the two parents is supplied as input. In this situation, the program will first focus on the polymorphisms detected between the two parents and proceed as usual.

In addition to the R, D, Pr and Pd samples, the program supports the inclusion of an additional wild-type sample, designated Wd or Wr, corresponding to an isogenic line or the non-mutagenized parents of a dominant mutant or a recessive mutant, respectively. The Wd and Wr samples are then used to discard any alleles that were already present prior to mutagenesis. The difference between P samples and W samples is that the former are used for determining the parental origin of alleles, whereas the latter are only used for discarding shared alleles.

### Analysis of QTL-seq data

In a similar way, the qtl command processes the VCF input data and calculates the necessary parameters for the mapping of QTLs using a QTL-seq strategy. At a minimum, it requires sequence data from two sets of individuals with extreme phenotypes, denoted H (‘high’) and L (‘low’), to calculate several parameters, including the ∆SNP index, *p*-values from Fisher’s exact tests, Euclidean distances for individual markers (ED_m_), and G statistics, which are commonly used in BSA-seq experiments [26]. This command supports the following scenarios:

(i) *Genomic DNA sequenced from the H and L bulks*. The AFs in the high and low bulks are used to calculate the ∆SNP index when the alleles can be classified based on their parental origin ( i.e., when the reference genome corresponds to one of the parents of the segregating population). If the parental origin of the alleles cannot be determined, the command reports the absolute value of the ∆SNP index (|∆SNP index|) in addition to the above statistics for each polymorphic site found.
(ii) *Genomic DNA isolated from the H and L bulks plus the resequencing of one parent (P).* In this case, the alleles can be assigned to haplotypes based on the sequence of the parent, allowing the calculation of ∆SNP indices and other parameters.

Because the qtl and mbs commands can receive input line-by-line from a Unix pipe, calculations that require data from multiple adjacent SNP markers are deferred to a later step, when they are performed by the merge and plot commands. This is the case for the calculation of ED100^4^ values (which are calculated as the fourth power of the sum of the Euclidean distances of 100 consecutive SNPs, and are plotted only for those chromosomes that contain enough markers), the calculation of moving averages of other parameters, or calculations that require knowing the number of statistical tests performed (e.g. significance thresholds corrected for multiple testing using the Bonferroni method).

### Binning of MBS and QTL-seq data

The merge command can be used to post-process the results generated by the mbs and qtl commands when the parental origin of each allele is known. This command should be particularly useful when the number of polymorphic sites is high (e.g. when using populations involving different genetic backgrounds) but the sequencing depth at each individual site is low, which might yield non-significant results at individual markers. In this case, we reasoned that binning the allele counts of adjacent markers can help to identify regions (rather than individual markers) linked to the trait of interest. Bins can be defined in two alternative ways: (a) as overlapping sets of *n* consecutive markers, or (b) as sets of all markers contained within non-overlapping windows of user-defined length. The read counts of all markers within a bin are aggregated and then used to calculate bin-level haplotype frequencies and other parameters relevant to MBS and QTL-seq experiments. When thousands of polymorphisms segregate in the mapping population and their linkage phase is known, we reasoned that increasing the number of observations would improve the ability of Fisher’s exact tests to detect a significant difference between treatments, since a larger sample size provides more information, reduces the impact of random variability, and increases the chances of detecting a true difference if it exists. An example of how the merge command affects the results of the analysis is shown below for Case Study 6.

### Plotting the results of MBS and QTL-seq experiments

The plot command is intended to generate publication-quality figures and to facilitate interpretation of the output of the mbs, qtl and merge commands. The set of plots produced can be customized by the user or automatically selected based on the fields present in the header of the output files produced by each command. These plots can be drawn for individual chromosomes or integrated into multi-panel figures: the user can select specific chromosomes and parameters to plot, and can choose to create figures that integrate different parameters for a given chromosome, or figures that display a given parameter for a user-selected set of chromosomes. *p*-values and other parameters can also be presented as Manhattan-like plots. Additional features of this command include the ability to plot moving averages of the allele frequencies (calculated either using all markers within overlapping windows containing a user-defined number of adjacent markers or using all markers within non-overlapping intervals of user-defined length) and ‘boost’ values (calculated as described for SHOREmap), the ability to overlay the moving average of the AF for the alternative pool, the automatic generation of figure legends, and the availability of different color palettes. As a proxy for a confidence interval for the ∆SNP-index plots, we average the limits of the confidence intervals calculated for individual markers. These limits are calculated based on the formulas for a difference of two proportions, applying the Bonferroni correction to the significance level used (𝛼=0.05), taking into account the number of markers. Similarly, the *p*-value plots incorporate a significance threshold level that is calculated using the Bonferroni correction. The user can customize the appearance of the plots by selecting appropriate options on the command line, including predefined color combinations (“palettes”), dot size and transparency, line width, resolution and output file format (EPS, JPG, PDF, PNG or SVG). The program allows users to customize additional aspects by editing a file in JavaScript Object Notation (JSON) format that MAPtools reads from disk. Parameters that can be customized include color palettes, some display attributes, and chromosome aliases. Although the program comes with a default color palette and a color palette optimized for individuals with color blindness, the JSON file also allows users to define their own custom palettes and adjust the size and other display attributes of the plots generated by MAPtools. By default, chromosomes are labeled as they appear in the reference genome’s FASTA file, typically using their GenBank accession numbers, but the JSON file allows users to assign a shorter, alternative display name to each chromosome (e.g., ‘Chr1’). The ability to generate files in vector graphics formats, such as the EPS (Encapsulated PostScript) and SVG (Scalable Vector Graphics) formats, provides an additional level of customization, allowing users to easily edit elements such as caption sizes and axis labels while ensuring that images are displayed at the maximum resolution.

### Functional annotation of identified variants

The annotate command assesses the functional impact of nucleotide substitutions, insertions, and deletions within a user-specified interval. Certain variants are typically excluded based on predefined criteria [27]: (a) the mutant allele matches the reference genome allele, which is assumed to be functional, (b) the substitution is not a G/C-to-A/T transition mutation (the most common type of mutation caused by ethyl methanesulfonate, EMS), or (c) the mutant allele is present in the non-mutagenized parent or other related lines, indicating that it does not cause the observed phenotype. We have implemented these filters in the mbs command, allowing the user to generate filtered or unfiltered input for annotate at will. The command then quickly evaluates the effect of all candidate mutations passing these filters against the genome annotation provided as a GFF3 file using a binary search algorithm.

This command identifies whether mutations reside in genic or intergenic regions. For intergenic mutations, it reports the identity of and the distance to the nearest adjacent genes. In protein-coding genes, the program determines whether the mutation is located in the 5’ untranslated region (5’-UTR), the coding sequence (CDS), or the 3’ untranslated region (3’-UTR). Substitutions in the CDS are classified as synonymous or non-synonymous (with the latter further classified as missense or nonsense) based on their effect on the amino acid sequence. Indels are checked for frameshift generation. For mutations located in introns, the program reports the distance to the nearest adjacent exons in each transcript isoform. Mutations near donor or acceptor splice sites that could cause missplicing are also reported. Mutations in the 5’-UTR are checked for premature ATG codon creation and their distance to the translation initiation site is reported. Nonstop mutations that replace a stop codon, resulting in continued mRNA translation into the 3’-UTR, are also reported. The program also assesses whether deletions partially or completely disrupt one or more genes.

### Validation

We have tested MAPtools under a variety of experimental situations, using our own MBS data [28], simulated MBS data, and publicly available MBS and QTL-seq datasets reported in the literature for plant species as diverse as Chinese cabbage (*Brassica rapa* L. ssp. *pekinensis*) [29, 30], rice (*Oryza sativa* L.) [6, 31, 32], tomato (*Solanum lycopersicum* L.) [33], strawberry (*Fragaria vesca* L.) [34, 35], and *Arabis alpina* [36]. As shown below, our results demonstrate the ability of MAPtools to analyze data across different experimental designs and species, as we were able to detect the same QTL and find the same causal mutations as reported in the published studies (Supplemental File 1). Table 1 and Supplemental Table 1 summarize the datasets that have been analyzed and the options used to run the MAPtools’ commands mbs and qtl.

**Table 1:** Case studies used for testing MAPtools

Species	Trait or mutation	BioProject (samples)	Reference
*O. sativa*	*lcd1*	PRJNA525315 (D: SRR8695238; R: SRR8695239; Pr: SRR8695240; Pd: SRR8695241)	Cao et al., 2019
*O. sativa*	Suppresor of *xantha*	PRJCA007389 (D: CRR344193; R: CRR344195; Pd: CRR344192; Pr: CRR344194)	Jiang et al., 2022
*O. sativa*	Resistance to rice blast disease	PRJDB2455 (H: DRR003237; L: DRR003238)	Takagi et al., 2013
*S. lycopersicum*	Ascorbate-enriched fruits (AsA+)		Bournonville et al., 2023
*B. rapa*	*nhm3*	PRJNA761522 (R: SRR15829494; Pd: SRR15803269; Pr: SRR15828094)	Huang et al., 2022
*B. rapa*	Cuticular wax biosynthesis	PRJNA751715 (D: SRR15371666, R: SRR15371667)	Yang et al., 2022
*A. alpina*	*eop002*	PRJNA756904 (R: SRR15564670) and PRJNA608065 (Pd: SRR11140832-SRR11140833)	Viñegra de la Torre et al., 2022
	*eop085*	PRJNA756904 (R: SRR15564669) and PRJNA608065 (Pd: SRR11140832-SRR11140833)	
	*eop091*	PRJNA756904 (R: SRR15564668) and PRJNA608065 (Pd: SRR11140832-SRR11140833)	
*A. thaliana*	*emb1956-3*	PRJNA9349:07 (D SRR23456103; R: SRR23456104) and PRJNA751183 (Wr: SRR15322352)	Rodríguez-Alcocer et al., 2023
*F. vesca*	Petiole color	PRJNA823731 (R: SRR18649835; D: SRR18649836)	Luo et al., 2023
*F. vesca*	Fruit color	PRJEB38128 (R: ERR4463155-ERR4463156; D: ERR4463153-ERR4463154)	Castillejo et al., 2020

Projects and samples with accession numbers are publicly available from the NCBI (http://ncbi.nlm.nih.gov) and Genome Sequence Archive BIG Data Center (https://bigd.big.ac.cn/gsa/) databases

**Fig. 2:**
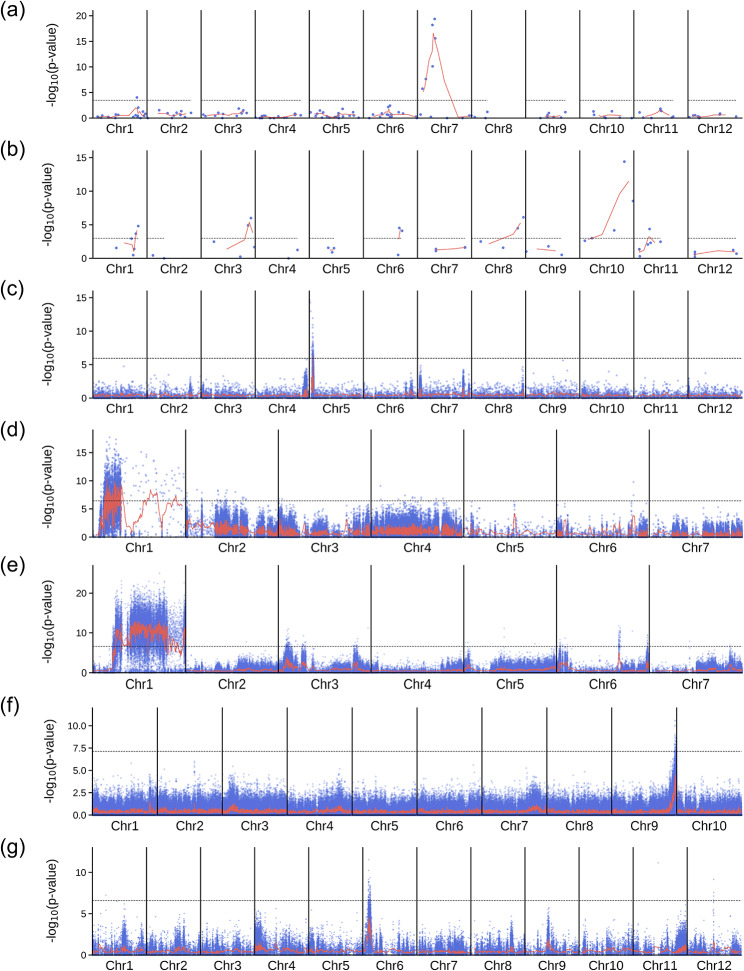
Mapping-by-sequencing in different species using MAPtools. The dots in each Manhattan plot correspond to the -log(*p*-value) of two-tailed Fisher’s exact tests performed for individual biallelic markers segregating in the mapping population, as determined using data from the R and D bulks. The lines correspond to the weighted moving averages calculated for a sliding window containing *n* adjacent markers. The dashed line marks the significance threshold calculated using the Bonferroni correction. **(a)** Mapping of the *lcd1* mutation of rice; *n* = 3. **(b)** Mapping of a suppressor of the *xantha* mutant of rice; *n* = 2. **(c)** Mapping of an ascorbate-enriched mutant of tomato; *n* = 20. **(d)** Mapping of the *green petiole-1* mutant of strawberry; *n* = 20. **(e)** Mapping of a white fruit mutant of strawberry; *n* = 100. **(f)** Mapping of a glossy mutant of Chinese cabbage; *n* = 100. **(g)** Mapping QTL for a rice blast disease; *n* = 100

### Case study 1: The *lcd1 *mutant of rice

**Fig. 3:**
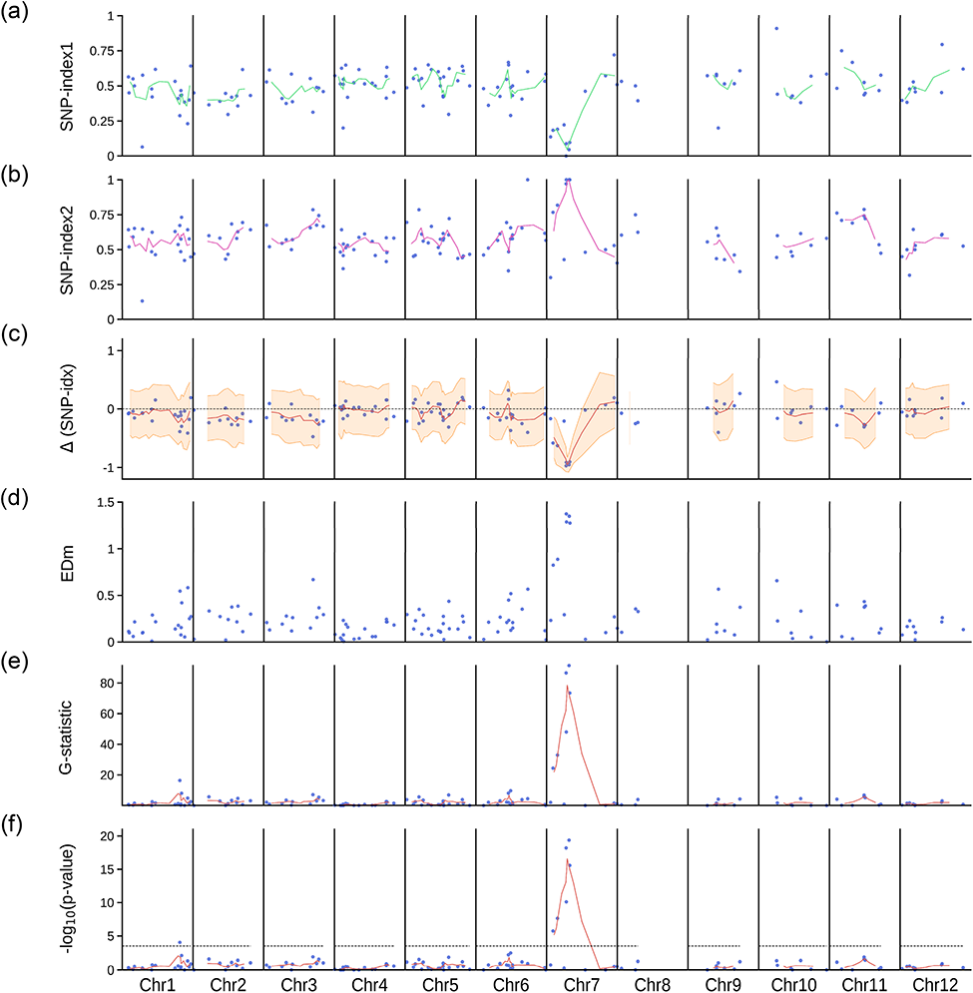
Mapping-by-sequencing of the *lcd1* mutant of rice. Several statistics have been evaluated across all chromosomes, and the results are presented as Manhattan plots. Each dot corresponds to an individual biallelic marker segregating in the population. Weighted moving averages (continuous lines) have been calculated for each statistic using a sliding window containing 3 adjacent markers. **(a)** SNP-index (allele frequency) in the D bulk. **(b)** SNP-index in the R bulk. **(c)** Δ(SNP-index), calculated as the difference between the SNP-index of the D bulk and the SNP-index of the R bulk. The shaded area is delimited by the moving averages of the lower and upper bounds of 95% confidence intervals, using the Bonferroni correction for multiple testing (with *n* = 151 tests). **(d)** Euclidean distance. **(e)** G-statistic, calculated as described by Magwene et al. (2011). **(f)** -log(*p*-value) of two-tailed Fisher’s exact tests. The dashed line marks the Bonferroni-corrected 5% significance threshold, calculated considering that *n* = 151 chromosomal locations have been tested

The recessive *lcd1* (*low Cd accumulation 1*) mutant was induced by EMS mutagenesis from 9311, an *indica* rice strain for which a reference genome sequence is available [31]. To map the gene, the authors used an F_2_ population derived from a backcross between *lcd1* and its wild-type progenitor, 9311, and used the Illumina platform to sequence four samples: the *lcd1* and 9311 parents of the cross, a bulk comprising the 31 F_2_ plants with the lowest Cd levels (presumably *lcd1* mutants), and another bulk comprising the 31 F_2_ plants with the highest levels. The known effects of EMS and the fact that both parents of the cross have been sequenced, allowing the alleles to be classified according to their parental origin, make this an ideal case study for testing MAPtools. We run the MAPtools mbs command with the -dR,D,Pr,Pd option, which instructs the program to use the sequence data from the parental samples (Pr and Pd) to select the variants that will be analyzed in the bulks of phenotypically dominant (D) and recessive (R) plants. An advantage of resequencing of the parents (Pr and Pd) is that each allele can be assigned to its parental haplotype. Alternatively, this assignment could have been made by selecting the -r D option, since the reference genome sequence corresponds to one of the parents of the mapping population (i.e. 9311). Our analysis of the original data allowed us to locate the gene on chromosome 7, as indicated by the significant *p*-values of Fisher’s exact tests (Fig. 2a) and all other parameters used to compare the distribution of alleles in the D and R bulks (Fig. 3 and Supplemental Fig. 1). By applying the annotate command to a 5-Mb candidate interval on chromosome 7, we were able to detect the same C-to-T transition mutation in exon 7 of the Os07g0257200 gene as previously described [31]. While these authors reported that the mutation substitutes a Leu residue for Pro in the OsNRAMP5 protein, MAPtools additionally indicated that the *lcd1* mutation damages a 5’ splice site and, therefore, it may disrupt the protein function to a greater extent than originally thought.

### Case study 2: a suppressor of a *xantha* mutant of rice

A similar approach was followed to characterize a mutation that suppresses the effects of a *xantha* (yellow-leaf) mutation in rice [32]. To generate a mapping population, the suppressor was backcrossed to the *xantha* mutant. Rather than focusing on the F_2_ generation, the authors bulked F_3_ plants from the generation that were known to be homozygous for either the mutant allele or the wild-type allele. In addition to the parents of the cross, two bulks were sequenced, one consisting of 20 F_2:3_ plants exhibiting the *xantha* phenotype and another consisting of 20 F_2:3_ plants with green leaves. Although MAPtools is not specifically designed to work with bulks of F_3_ plants, it allowed us to successfully locate the gene on chromosome 10 (Fig. 2b). The G-to-A transition reported in the article [32] was the only mutation detected by the annotate command within a 3 Mb candidate interval. The command reported its effect on three isoforms of the Os10g0502400 (OsGluTR) gene. In one isoform, the mutation is predicted to reside in the 5’ untranslated region (5’ UTR) and its effect is uncertain. In the other isoforms, however, the mutation is located in the CDS and is predicted to substitute a Val residue for Ala in the corresponding protein products. Interestingly, this was the only position reported by the program for which the two bulks were homozygous, albeit for different alleles, as expected from experimental design (Supplemental Figs. 2 and 3).

### Case study 3: an ascorbate-enriched mutant of tomato

We also analyzed raw data corresponding to a recessive mutant with ascorbate-enriched fruits that had been isolated from the tomato cultivar Micro-Tom after EMS mutagenesis [33]. The data from this study allowed us to test MAPtools when the genome of one parent has been resequenced (i.e. Micro-Tom) in addition to the D and R bulks. Running the mbs command with the -d D,R,Pd option allowed us to place the gene on chromosome 5 (Fig. 2c and Supplemental Figs. 4 and 5). Using the annotate command, we found a C-to-T transition mutation in the gene Solyc05g007020, which encodes a member of the PAS/LOV protein (PLP) class of photoreceptors. The authors correctly reported that this mutation creates a premature stop codon [33], but the annotate command additionally reported that it damages a 5’ splice site and is therefore likely to alter the splicing of its transcripts.

### Case study 4: the *nhm3-1* mutant of Chinese cabbage

**Fig. 4:**
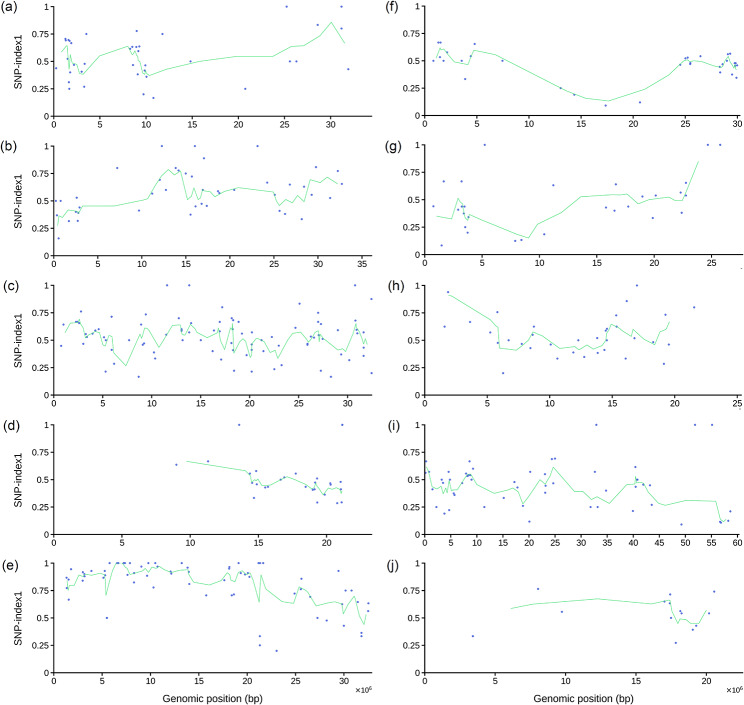
Allele frequencies place the *nhm3-1* mutation on chromosome 5 of Chinese cabbage. Each dot indicates the allele frequency of a biallelic polymorphism segregating in the population, as determined for the R bulk. The light green line indicates the moving average of the allele frequencies at 3 adjacent sites. **(a)** Chromosome A01. **(b)** Chromosome A02. **(c)** Chromosome A03. **(d)** Chromosome A04. **(e)** Chromosome A05. **(f)** Chromosome A06. **(g)** Chromosome A07. **(h)** Chromosome A08. **(i)** Chromosome A09. **(j)** Chromosome A10

A different approach was followed to characterize a recessive *non-heading mutant* (*nhm3-1*), which had been induced by EMS from FT, a wild-type strain of Chinese cabbage (*Brassica rapa* ssp. *pekinensis*) [30]. The authors prepared an F_2_ mapping population, but they chose to sequence only the bulk of F_2_ mutant plants (R bulk) and the two parents of the population (FT and the *nhm3-1* mutant). To handle this situation, we run MAPtools mbs with the -d R,Pd,Pr option. In the absence of a D bulk, we rely entirely on the allele frequencies to map and identify the mutation, as MAPtools cannot perform any calculations that require the two bulks (e.g. Fisher’s exact tests, G statistics or Euclidean distances). The allele frequencies in the R bulk indicate that the *nhm3-1* mutation is most likely located on chromosome 5 (Fig. 4). We used the annotate command to evaluate the effect of the 20 EMS-type nucleotide substitutions detected in a wide candidate interval (between megabases 3 and 10) on this chromosome. Interestingly, all the substitutions were of the same type (G-to-A transitions, with no C-to-T transitions detected in the interval), a bias that is a known consequence of the mutagenic action of EMS on the same DNA strand [37]. One of the transition mutations found by the annotate command is predicted to cause a Gly-to-Glu substitution in the protein encoded by the *KAO2* (*ent*-kaurenoic acid oxidase 2) gene, also known as A05p015130.1_BraROA.1. This mutation was previously considered to be the most likely cause of the observed phenotype [30]. However, the candidate interval was found to contain a second nonsynonymous mutation with an allele frequency of 1 in the A05p016250.1_BraROA.1 gene, which is predicted to cause a Gly-to-Asp substitution in the protein.

### Case study 5: three *eop* mutants of *Arabis alpina*

**Fig. 5:**
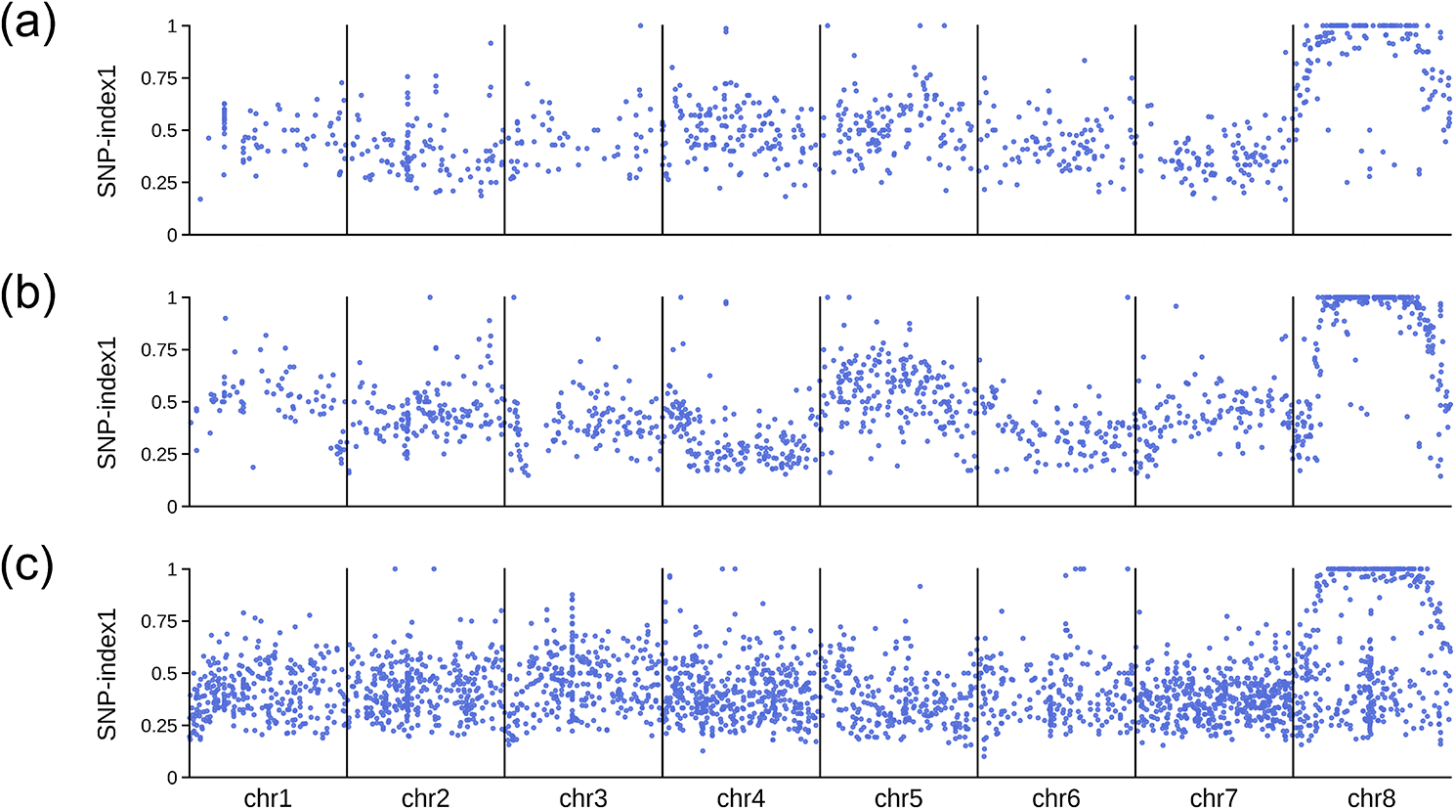
Mapping-by-sequencing of three recessive *eop* mutants of *Arabis alpina*. Each dot corresponds to an individual biallelic marker segregating in the population.**(a) ***eop002* mutant. **(b) ***eop085* mutant. **(c) ***eop091* mutant

Viñegra de la Torre et al. [36] followed a mapping-by-sequencing strategy to characterize three EMS-induced alleles (*eop002*, *eop085* and *eop091*) of the *ENHANCERS OF PEP1* (*EOP*) gene of *Arabis alpina*. The authors prepared mapping populations for the three mutants and sequenced one bulk of F_2_ mutant plants for each one, plus an additional sample of the *pep1-1* parent of the three populations. All four samples were sequenced using Illumina technology, and the reads were mapped to the *A. alpina* reference genome. We used the MAPtools mbs command with the -d R,Pd option to map the three allelic mutations separately. The allele frequencies in the R bulk of each mapping population clearly indicated that the mutations reside on chromosome 8 (Fig. 5 and Supplemental Fig. 6). Using annotate, we were able to identify three nonsynonymous mutations affecting the same gene (Aa_G106560) on this chromosome, as had been previously reported.

### Case study 6: an albino mutant of *Arabidopsis thaliana*

**Fig. 6:**
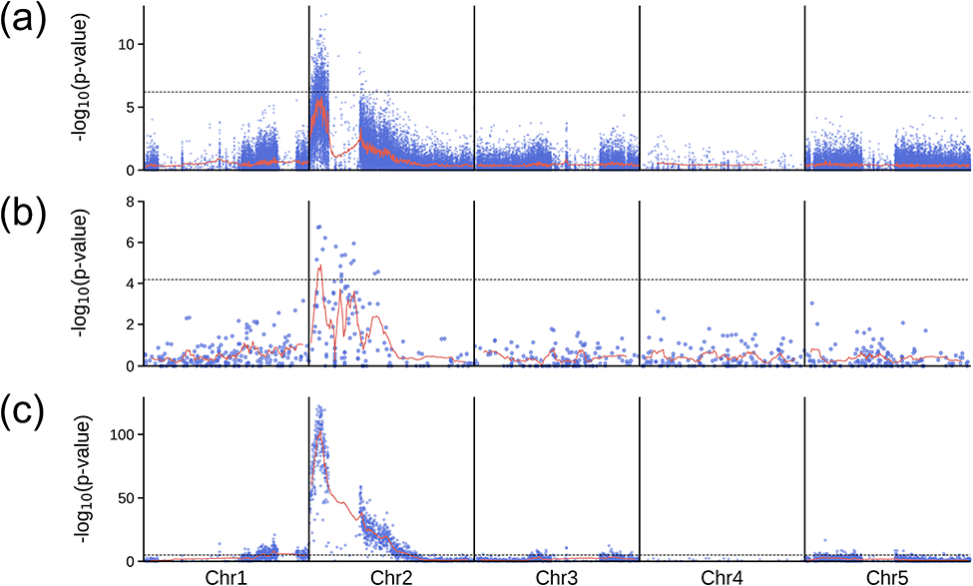
Mapping-by-sequencing of an albino mutant of *A. thaliana*. Each dot in the Manhattan plots corresponds to the -log(*p*-value) of a two-tailed Fisher’s exact test performed for an individual biallelic marker segregating in the population (**a** and **b**) or for haplotypes integrating the allele counts of 20 adjacent markers (**c**), as determined using data from the R and D bulks. The panels illustrate the effect of running the mbs and merge commands of MAPtools on the same dataset with different options. **(a)**mbs -d D,R -m R -r D, with no filters applied. **(b)**mbs -d D,R,Wr -r D -m R --EMS -I --parental-filter, which excludes indels (-I), all mutations other than G/A-to-C/T transitions (--EMS), and all changes already present in the Wr sample (--parental-filter). **(c)**mbs -d D,R -m R -r D, followed by merge -w 20, which combines the allele counts in sets of 20 consecutive markers. The lines correspond to the weighted moving averages calculated for a sliding window containing 200 markers (**a**), 5 markers (**b**) and 10 haplotypes (**c**). The dashed lines mark the Bonferroni-corrected 5% significance thresholds, assuming that *n* = 291,824 (a), *n* = 861 (b) and *n* = 14,595 tests have been performed

To test MAPtools in the context of mapping populations derived from crosses involving widely divergent genetic backgrounds, we have also reanalyzed previously published data from several articles, including our own. We recently described the cloning of an albino mutation in *Arabidopsis thaliana* using mapping by sequencing [28]. In this particular case, we had sequenced two bulks of plants from the F_2_ generation, one consisting of 170 phenotypically wild-type plants and another consisting of 87 phenotypically mutant plants. To analyze the raw data from this experiment, we used the MAPtools mbs command with the -d D,R option, which allowed us to locate the mutation on chromosome 2 (Fig. 6a and Supplemental Figs. 7 and 8). The resulting plots revealed that the genomes of the two parental strains consisted of regions that differed in the abundance of sequence polymorphisms. To exclude additional polymorphisms that were unlikely to cause the observed phenotype, we reran the command with the --EMS and -I flags, which discard substitutions that are unlikely to have been caused by EMS as well as all insertion-deletion mutations: This second analysis was performed with the -d D,R,Wr and --parental-filter options. To this end, we added sequencing data for wild-type plants of the Landsberg *erecta* background, which shares polymorphisms with the mutant parent of the mapping population. These filters greatly reduced the number of candidate mutations (Fig. 6b) and facilitated the identification of the same causal mutation as previously reported [28]. To illustrate the effect of the merge command, we applied it to the unfiltered dataset, which resulted in a more pronounced peak in the Manhattan plots (Fig. 6c).

#### Case study 7: the *green petioles-1* mutant of strawberry

In addition to our own data, we have also tested the program with data from outcrosses involving plant species other than *Arabidopsis thaliana*. A recent study has characterized the *green petioles-1* (*gp-1*) mutant of diploid strawberry (*Fragaria vesca*), which was isolated after *N*-ethyl-*N*-nitrosourea (ENU) mutagenesis of the Yellow Wonder 5AF7 (YW) accession [35]. The petioles of wild-type plants are purple whereas those of *gp-1* mutants are green. An F_2_ population derived from an outcross between the *gp-1* mutant and the Hawaii (H4) wild-type accession was used for mapping-by-sequencing. Two bulks were sequenced: one consisting of F_2_ plants with green petioles, and another consisting of F_2_ plants with purple petioles. Since the genome sequence of the H4 accession is available and was used as the reference for mapping the reads, MAPtools can easily assign the alleles to the two parents of the outcross. With the -d R,D -r D -m R options, the allele frequency and *p*-value plots generated by MAPtools suggested that the mutation resides on chromosome 1 (Fig. 2d and Supplemental Figs. 9 and 10). Using the annotate command, we identified the same C-to-T substitution on chromosome 1 as previously described [35]. Based on the annotation provided in the GFF file, MAPtools determined that it is located one nucleotide away from a 3’ splice site within one of the introns of the FveMYB10L gene (FvH4_1g22040.1). Luo et al. [35], however, corrected the annotation of this gene so that the mutation is located in exon 3, resulting in the substitution of a lysine reside for an arginine in the protein.

### Case study 8: a white fruit mutant of strawberry

Castillejo et al. [34] mapped a mutation causing white fruits using two bulks from the F_2_ progeny of an outcross involving two lines of strawberry (*Fragaria vesca*), RV660 (with red fruits) and WV596 (with white fruits). The bulks comprised 34 plants with red fruits and 32 plants with white fruits, respectively. The reads were aligned to the Hawaii-4 (H4) reference genome and, because the parents of the mapping population were not sequenced, their alleles cannot be uniquely assigned to either RV660 or WV596. Using the ΔSNP index, the authors defined a candidate interval on chromosome 1, where they identified a transposon insertion in the *FvMYB10* gene (FvH4_1g22020). Our analysis of the raw data using the MAPtools mbs command clearly showed that the mutation responsible for the white color resides on chromosome 1, as evidenced by the low *p*-values of Fisher’s exact tests (Fig. 2e) and other parameters considered, such as the frequency of the most abundant allele (Supplemental Figs. 11 and 12). Although the annotate command is not designed to detect large insertions, like the transposon reported by Castillejo et al. (2020), it identified other polymorphisms with highly skewed allele counts in the coding sequence of the same gene (i.e. a G-to-A transition mutation at position 13,950,746).

### Case study 9: a glossy mutant of Chinese cabbage

In another study with Chinese cabbage [29], the genetic basis of the recessive glossy phenotype of a line was characterized. To identify the responsible locus, a BSA-seq strategy was followed with an F_2_ mapping population derived from the cross between the glossy line (Y1211-1) and a double haploid line (R16-11). Two pools of F_2_ plants were sequenced: one consisting of 25 glossy plants (the G pool) and one consisting of 25 waxy plants (the W pool). The authors aligned the reads to the *B. rapa* v1.5 genome, which did not match either parent of the cross. By examining Δ(SNP index) values, they found a candidate gene, Bra032670, on chromosome A09, in a candidate interval between megabases 37.35 and 38.88. This study is based on an isogenic cross, but the parents are not available to sort the alleles (neither reference sequence nor additional sequenced pools). Using MAPtools, a maximum of allele frequencies and a minimum of *p*-value in the same chromosome as indicated by the authors of the paper are clearly detected (Fig. 2f and Supplemental Figs. 13 and 14).

### Case study 10: mapping QTL for rice blast disease

Takagi et al. [6] mapped QTL conferring partial resistance to rice blast disease (*Magnaporthe oryzae*) using a population of recombinant inbred lines (RILs) established from a cross between the Nortai (partially resistant) and Hitomebore (highly susceptible) rice lines. The RILs were scored for resistance, and two bulks were made with 20 lines highly resistant and 20 lines highly susceptible to rice blast. The reads were mapped to the Hitomebore reference genome, which allows the assignment of alleles to each parent. Using MAPtools, we mapped a QTL to a relatively narrow region on chromosome 6, at the same location reported by Takagi et al. (2013), as indicated by the significant *p*-values of Fisher’s exact tests (after Bonferroni correction) and the shift in the value of the ΔSNP indices (Fig. 2g and Supplemental Figs. 15 and 16).

## Conclusions

Here, we present MAPtools, a novel program with a number of useful features for the analysis and visualization of mapping-by-sequencing and QTL-seq data. This command-line tool is implemented in the Python3 language, making it easy to install and use. We developed MAPtools inspired by the SAMtools and BCFtools packages, two important applications that we emulated in features such as the availability of distinct commands and the ability to receive input data through a command-line pipeline or properly formatted VCF files. The fact that MAPtools can process input received from the command line makes it a very versatile application that can be easily integrated into workflows with various state-of-the-art variant callers, such as BCFtools or GATK. Although the mbs and qtl commands of MAPtools primarily function with VCF input data, this feature of the program effectively allows processing input data in BCF format by simply adding a conversion step (e.g. by using BCFtools’ view command) to the workflow. Once a VCF (BCF) file is ready, it can be quickly processed any number of times by running MAPtools mbs or qtl with user-selected parameters to facilitate the identification of the causal mutation, or by adding additional steps to the workflow. These may include steps to filter out sites with certain types of mutations (e.g. indels), too low sequencing depth, or based on the values of other fields present in the VCF file format.

One of the unique features of MAPtools is its ability to use multiple criteria to determine the position of QTLs or genes of interest. In addition, our repertoire of commands enables the automatic generation of publication-quality figures and their captions, as well as the rapid assessment of the functional impact of identified genetic variants by using a genome annotation file in the GFF3 standard format. We have tested our software using raw data from species with genome sizes ranging from 135 Mbp (*A. thaliana*) to 828 Mbp (*S. lycopersicum*). MAPtools will work best for any species for which a high-quality genome sequence is available, particularly if it has been annotated using the standard GFF3 file format. To this end, the Ensembl Plants database turned out to be an ideal resource [38], as it includes genomes for over 100 plant species, which opens the door to a wide range of potential uses for MAPtools. While in most cases we mapped the reads to genomes downloaded from Ensembl Plants, we also obtained satisfactory results with genomes from different sources (e.g. for *F. vesca*). Importantly, the Ensembl database also includes the genomes of many different organisms to which the MBS or QTL-seq methodology could also be applied [39]. We plan to expand the repertoire of commands available in MAPtools to enable the analysis of data from other experimental scenarios involving massively parallel sequencing, such as the construction of high-resolution linkage maps.

### Electronic supplementary material

Below is the link to the electronic supplementary material.

Supplementary Material 1

Supplementary Material 2

Supplementary Material 3

## Data Availability

The program and documentation are available from https://github.com/hcandela/MAPtools.
