# Supplementary material for: MAPtools: command-line tools for mapping-by-sequencing and QTL-Seq analysis and visualization
Source: Plant Methods. 2024 Jul 17;20:107. doi: 10.1186/s13007-024-01222-2 (PMC11253474; doi:10.1186/s13007-024-01222-2)
Supplement: Supplementary file 2 — Supplementary Material 2 [file 13007_2024_1222_MOESM2_ESM.pdf]

Supplemental File 2: MAPtools commands used in each case study.

This document outlines the steps followed in the analysis of all case studies after preparing the sorted BAM files for all the available samples.

Cao 2019:

variant calling

```
bcftools mpileup --threads 20 -f
Oryza_indica.ASM465v1.dna.toplevel.fa -I --annotate FORMAT/AD
SRR8695238.bam SRR8695239.bam SRR8695240.bam SRR8695241.bam |
bcftools call -mv -O b -o oryza.bcf –
```

running the mbs command

```
bcftools view oryza.bcf | ./maptools.py mbs -d D,R,Pr,Pd -m R --
parental-filter -I --EMS -o mbs_oryza.txt
```

running the plot command

```
./maptools.py plot -i mbs_oryza.txt -A 3 --captions  --
bonferroni --ci95 -t 0.8 -D 5 -a -m -O jpg -o plots_oryza
```

running the annotate command

```
./maptools.py annotate -i mbs_oryza.txt  -g
Oryza_indica.ASM465v1.57.chr.gff3 -f
Oryza_indica.ASM465v1.dna.toplevel.fa -R 7:6800000-9200000 -o
annotate_oryza.txt
```

Jiang 2022:

variant calling

```
bcftools mpileup --threads 20 -f Oryza_sativa.IRGSP-
1.0.dna.toplevel.fa -I --annotate FORMAT/AD CRR344193.bam
CRR344195.bam CRR344192.bam CRR344194.bam | bcftools call -mv -O
b -o oryza2.bcf –
```

running the mbs command

```
bcftools view oryza2.bcf | ./maptools.py mbs -d D,R,Pd,Pr -m R -
-parental-filter -c 8 -o mbs_oryza2.txt
```

running the plot command

```
./maptools.py plot -i mbs_oryza2.txt -A 3 --captions  --
bonferroni --ci95 -t 0.8 -D 5 -a -m -O jpg -o plots_oryza2
```

running the annotate command

```
./maptools.py annotate -i mbs_oryza2.txt -f Oryza_sativa.IRGSP-
1.0.dna.toplevel.fa -g Oryza_sativa.IRGSP-1.0.57.chr.gff3 -R
10:17000000-20000000 -o annotate_oryza2.txt
```

Bournonville 2023:

variant calling

```
bcftools mpileup --threads 20 -f sequenceMicro-Tom.fasta --
annotate FORMAT/AD P21H6_WT_PACBIO_sorted2.bam
P21H6_mut_PACBIO_sorted2.bam | bcftools call -mv -O b -o
microtom.bcf –
```

running the mbs command

```
bcftools view microtom.bcf | ./maptools.py mbs -d D,R,Pd -m R --
EMS -I --parental-filter -c 8 -o mbs_microtom.txt
```

running the plot command

```
./maptools.py plot -i mbs_microtom.txt --captions  --bonferroni
--ci95 -a -m -O jpg -o plots_microtom
```

running the annotate command

```
./maptools.py annotate -i mbs_microtom.txt -g
Solanum_lycopersicum.SL3.0.56.gff3 -f
Solanum_lycopersicum.SL3.0.dna.toplevel.fa -R 5:0-5000000 -o
annotate_microtom.txt
```

Huang 2022:

variant calling

```
bcftools mpileup -f
Brassica_rapa_ro18.SCU_BraROA_2.3.dna.toplevel.fa --annotate
FORMAT/AD SRR15829494.bam SRR15803269.bam SRR15828094.bam |
bcftools call -mv -O b -o brassica.bcf –
```

running the mbs command

```
bcftools view brassica.bcf | ./maptools.py mbs -d R,Pd,Pr -m R -
-parental-filter -I --EMS -o mbs_brassica.txt
```

running the plot command

```
./maptools.py plot -i mbs_brassica.txt -A 3 --captions  --
bonferroni --ci95 -t 0.8 -D 5 -a -m -O jpg -o plots_brassica
```

running the annotate command

```
./maptools.py annotate -i mbs_brassica.txt -R A05:3000000-
10000000 -f Brassica_rapa_ro18.SCU_BraROA_2.3.dna.toplevel.fa -g
Brassica_rapa_ro18.SCU_BraROA_2.3.57.chr.gff3 -o
annotate_brassica.txt
```

Yang 2022:

variant calling

```
bcftools mpileup --threads 20 -f
Brassica_rapa.Brapa_1.0.dna.toplevel.fa -I --annotate FORMAT/AD
SRR15371666.bam SRR15371667.bam | bcftools call -mv -O b -o
brassica2.bcf –
```

running the mbs command

```
bcftools view brassica2.bcf | ./maptools.py mbs -d D,R -m R -I -
-het-filter -q 10 -Q 90 -o mbs_yang_final.txt
```

running the plot command

```
./maptools.py plot -i mbs_brassica2.txt --captions  --bonferroni
--ci95 -a -m -O jpg -o plots_brassica2
```

running the annotate command

```
./maptools.py annotate -i mbs_brassica2.txt -f
Brassica_rapa.Brapa_1.0.dna.toplevel.fa -g
Brassica_rapa.Brapa_1.0.57.chr.gff3 -R A09:33000000-40000000 -o
annotate_brassica2.txt
```

Viñegra de la Torre 2022:

variant calling

```
bcftools mpileup -f Arabis_alpina.MPIPZ.V5.chr.all.fasta --
annotate FORMAT/AD eop085.bam pep1.bam | bcftools call -mv -O b
-o eop085.bcf –
```

```
bcftools mpileup -f Arabis_alpina.MPIPZ.V5.chr.all.fasta --
annotate FORMAT/AD eop091.bam pep1.bam | bcftools call -mv -O b
-o eop091.bcf –
```

```
bcftools mpileup -f Arabis_alpina.MPIPZ.V5.chr.all.fasta --
annotate FORMAT/AD eop002.bam pep1.bam | bcftools call -mv -O b
-o eop002.bcf –
```

The following steps were applied separately to each bcf file:

running the mbs command

```
bcftools view eop085.bcf | ./maptools.py mbs -d R,Pd -m R --
parental-filter --EMS -o mbs_eop085.txt
```

running the plot command

```
./maptools.py plot -i mbs_eop085.txt -A 3 --captions  --
bonferroni --ci95 -t 0.8 -D 5 -a -m -O jpg -o plots_eop085
```

running the annotate command

```
./maptools.py annotate -i mbs_eop085.txt -g
Arabis_alpina.MPIPZ.version_5.chr.all.liftOverV4.v3.gff3 -f
Arabis_alpina.MPIPZ.V5.chr.all.fasta -R chr8:14000000-35000000 -
o annotate_eop085.txt
```

Rodríguez-Alcocer 2023:

variant calling

```
bcftools mpileup -f Arabidopsis_thaliana.TAIR10.dna.toplevel.fa
--annotate FORMAT/AD G3clipped.bam G2clipped.bam G6control.bam |
bcftools call -mv -O b -o arabidopsis.bcf –
```

The mbs command was applied with or without the parental filter

running the mbs command with parental filter

```
bcftools view arabidopsis.bcf | ./maptools.py mbs -d D,R,Wr -m R
-r D --EMS --parental-filter -o mbs_arabidopsis.txt
```

running the mbs command without parental filter

```
bcftools view arabidopsis.bcf | ./maptools.py mbs -d D,R,Wr -m R
-r D --EMS -o mbs_arabidopsis2.txt
```

Running merge on the output of mbs (without parental filter):

```
./maptools.py merge -i mbs_arabidopsis2.txt -w 20 -o
mbs_arabidopsis_merged_20.txt
```

The following steps were applied separately to the output files of mbs and merge (using the corresponding file names):

running the plot command

```
./maptools.py plot -i mbs_arabidopsis.txt --captions  --
bonferroni --ci95 -a -m -O jpg -o plots_arabidopsis
```

running the annotate command (not applied to the output of merge)

```
./maptools.py annotate -i mbs_arabidopsis.txt -R 2:0-5000000 -f
Arabidopsis_thaliana.TAIR10.dna.toplevel.fa -g
Arabidopsis_thaliana.TAIR10.55.gff3 -o annotate_arabidopsis.txt
```

Luo 2023:

variant calling

```
bcftools mpileup -f Fragaria_vesca_v4.0.a1.normalized.fasta --
annotate FORMAT/AD SRR18649836.bam SRR18649835.bam | bcftools
call -mv -O b -o fragaria.bcf –
```

running the mbs command

```
bcftools view fragaria.bcf | ./maptools.py mbs -d R,D -m R -r D
-I -c 8 -o mbs_fragaria.txt
```

running the plot command

```
./maptools.py plot -i mbs_fragaria.txt --captions  --bonferroni
--ci95 -a -m -O jpg -o plots_fragaria
```

running the annotate command

```
./maptools.py annotate -i mbs_fragaria.txt -R Fvb1:12000000-
15000000 -f Fragaria_vesca_v4.0.a1.fasta -g
Fragaria_vesca_v4.0.a1.transcripts.gff3 -o annotate_fragaria.txt
```

Castillejo 2020:

variant calling

```
bcftools mpileup -f Fragaria_vesca_v4.0.a1.normalized.fasta --
annotate FORMAT/AD bam_white.bam bam_red.bam > fragaria2.vcf
```

running the mbs command

```
./maptools.py mbs -i fragaria2.vcf -d R,D -m R --het-filter -q
10 -Q 90 -o mbs_fragaria2.txt
```

running the plot command

```
./maptools.py plot -i mbs_fragaria2.txt --captions  --bonferroni
--ci95 -a -m -O jpg -o plots_fragaria2
```

running the annotate command

```
./maptools.py annotate -i mbs_fragaria2.txt -f
Fragaria_vesca_v4.0.a1.fasta -g
Fragaria_vesca_v4.0.a1.transcripts.gff3 -R Fvb1:7000000-15000000
-o annotate_fragaria2.txt
```

Takagi 2013:

variant calling

```
bcftools mpileup -f hitomebore_complete_genome.fasta -I --
annotate FORMAT/AD DRR003237.bam DRR003238.bam > hitomebore.vcf
```

running the qtl command

```
./maptools.py qtl -i hitomebore.vcf -d H,L -o qtl_hitomebore.txt
```

running the plot command

```
./maptools.py plot -i qtl_hitomebore.txt --captions  --
bonferroni --ci95 -a -m -O jpg -o plots_hitomebore
```
