# Supplementary material for: MAPtools: command-line tools for mapping-by-sequencing and QTL-Seq analysis and visualization
Source: Plant Methods. 2024 Jul 17;20:107. doi: 10.1186/s13007-024-01222-2 (PMC11253474; doi:10.1186/s13007-024-01222-2)
Supplement: Supplementary file 3 — Supplementary Material 3 [file 13007_2024_1222_MOESM3_ESM.pdf]

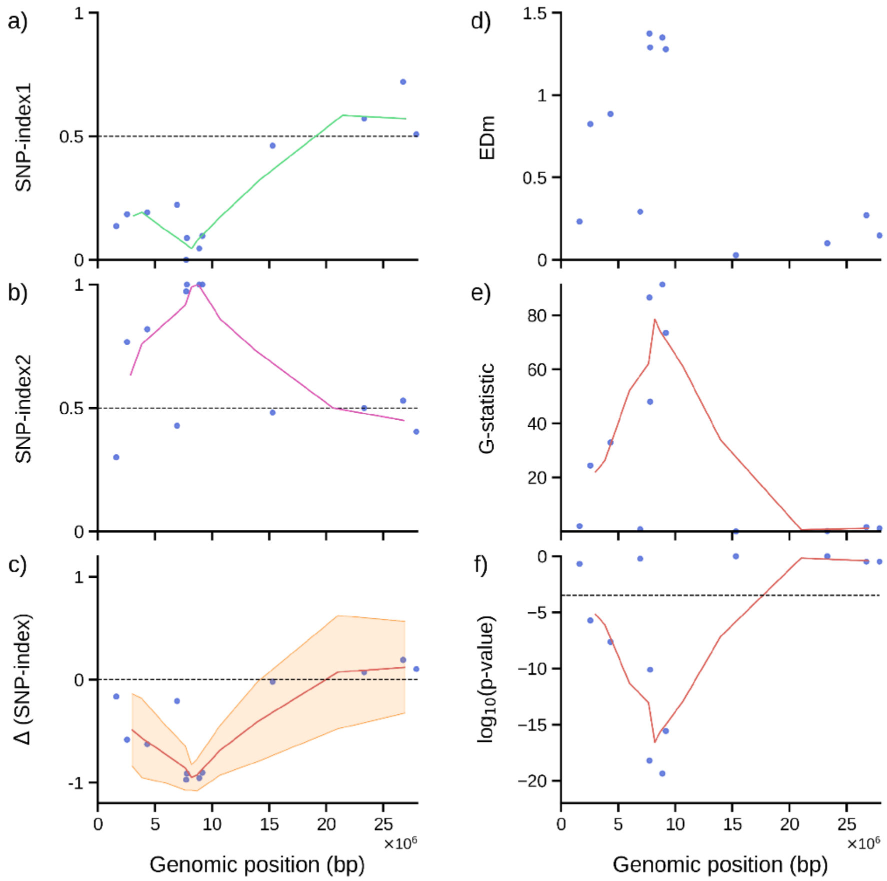

Supplemental Figure 1. Different statistics place the *lcd1* mutation on rice chromosome 7. Each dot corresponds to an individual biallelic marker segregating in the population. Weighted moving averages (continuous lines) have been calculated for each statistic using a sliding window containing 3 adjacent markers. **(a)** SNP-index (allele frequency) in the D bulk. **(b)** SNP-index in the R bulk. **(c)** Δ(SNP-index), calculated as the difference between the SNP-index of the D bulk and the SNP-index of the R bulk. The shaded area is delimited by the moving averages of the lower and upper bounds of 95% confidence intervals, using the Bonferroni correction for multiple testing (with n=151 tests). **(d)** Euclidean distance. **(e)** G-statistic, calculated as described by Magwene et al. (2011). **(f)** *p*-values of two-tailed Fisher's exact tests. The dashed line marks the Bonferroni-corrected 5% significance threshold, calculated considering that n=151 chromosomal locations have been tested.

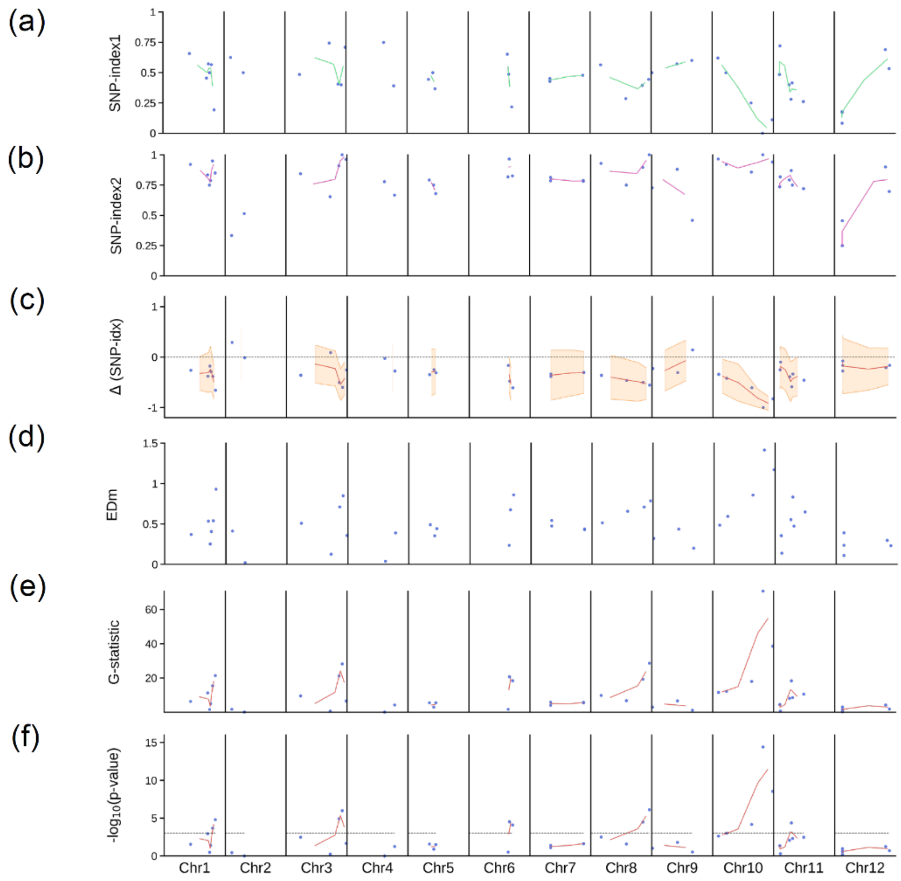

Supplemental Figure 2. Mapping-by-sequencing of a mutation that suppresses the *xantha* phenotype in rice. Several statistics have been evaluated across all chromosomes, and the results are presented as Manhattan plots. Each dot corresponds to an individual biallelic marker segregating in the population. Unless otherwise stated, continuous lines represent weighted moving averages calculated using a sliding window containing 2 adjacent markers. **(a)** SNP-index (allele frequency) in the D bulk. **(b)** SNP-index in the R bulk. **(c)** Δ(SNP-index), calculated as the difference between the SNP-index of the D bulk and the SNP-index of the R bulk. The shaded area is delimited by the moving averages of the lower and upper bounds of 95% confidence intervals, using the Bonferroni correction for multiple testing (with n=49 tests). **(d)** Euclidean distance. **(e)** G-statistic, calculated as described by Magwene et al. (2011). **(f)** -log(*p*-value) of two-tailed Fisher's exact tests. The dashed line marks the Bonferroni-corrected 5% significance threshold, calculated considering that n=49 chromosomal locations have been tested.

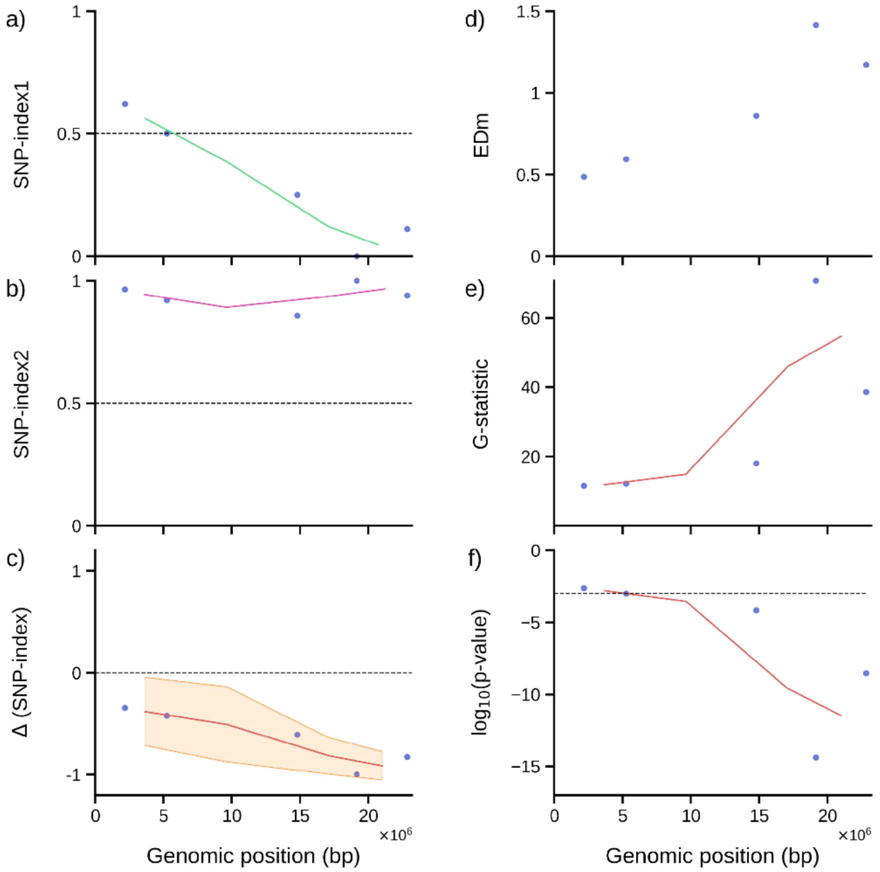

Supplemental Figure 3. Different statistics place a suppresor of *xantha* on rice chromosome 10. Each dot corresponds to an individual biallelic marker segregating in the population. Weighted moving averages (continuous lines) have been calculated for each statistic using a sliding window containing 2 adjacent markers. Unless otherwise stated, continuous lines represent weighted moving averages calculated using a sliding window containing 49 adjacent markers. **(a)** SNP-index (allele frequency) in the D bulk. **(b)** SNP-index in the R bulk. **(c)** Δ(SNP-index), calculated as the difference between the SNP-index of the D bulk and the SNP-index of the R bulk. The shaded area is delimited by the moving averages of the lower and upper bounds of 95% confidence intervals, using the Bonferroni correction for multiple testing (with n=670,109 tests). **(d)** Euclidean distance. **(e)** G-statistic, calculated as described by Magwene et al. (2011). **(f)** *p*-values of two-tailed Fisher's exact tests. The dashed line marks the Bonferroni-corrected 5% significance threshold, calculated considering that n=49 chromosomal locations have been tested.

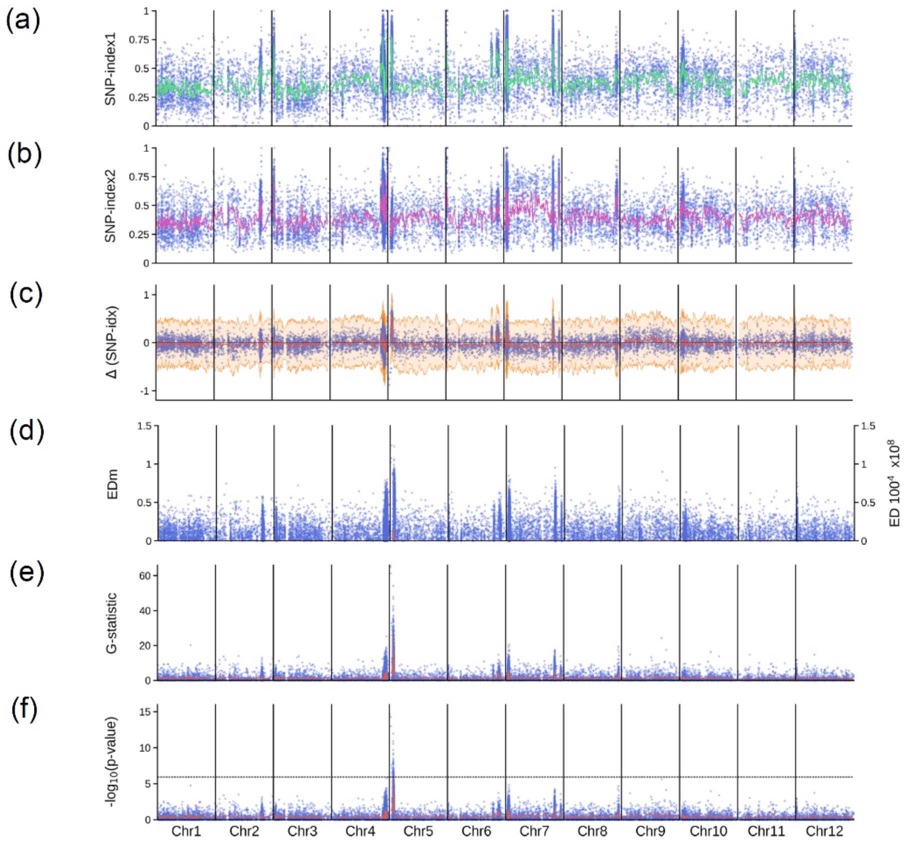

Supplemental Figure 4. Mapping-by-sequencing of an ascorbate-enriched mutant of tomato. Several statistics have been evaluated across all chromosomes, and the results are presented as Manhattan plots. Each dot corresponds to an individual biallelic marker segregating in the population. Unless otherwise stated, continuous lines represent weighted moving averages calculated using a sliding window containing 20 adjacent markers. **(a)** SNP-index (allele frequency) in the D bulk. **(b)** SNP-index in the R bulk. **(c)** Δ(SNP-index), calculated as the difference between the SNP-index of the D bulk and the SNP-index of the R bulk. The shaded area is delimited by the moving averages of the lower and upper bounds of 95% confidence intervals, using the Bonferroni correction for multiple testing (with n=41,415 tests). **(d)** Euclidean distance (dots) and $ED100^4$ (red line). $ED100^4$ values were calculated as described by de la Fuente Cantó et al. (2022). **(e)** G-statistic, calculated as described by Magwene et al. (2011). **(f)** -log(*p*-value) of two-tailed Fisher's exact tests. The dashed line marks the Bonferroni-corrected 5% significance threshold, calculated considering that n=41,415 chromosomal locations have been tested.

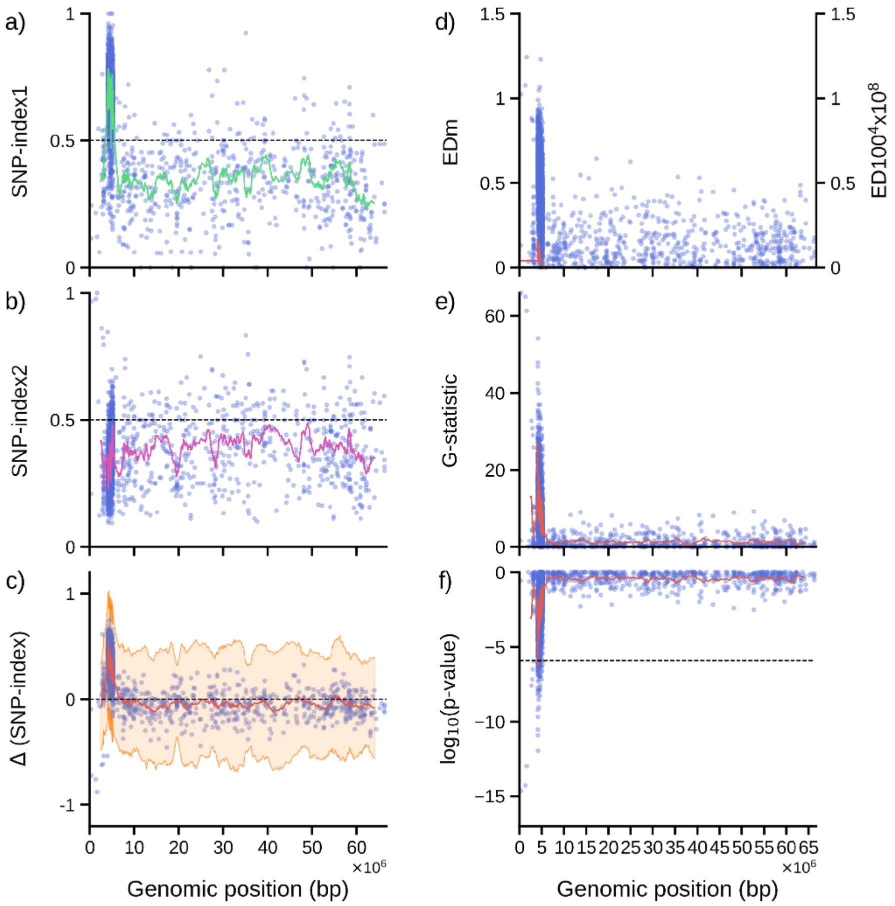

Supplemental Figure 5. Different statistics place an ascorbate-enriched mutation on tomato chromosome 5. Each dot corresponds to an individual biallelic marker segregating in the population. Unless otherwise stated, continuous lines represent weighted moving averages calculated using a sliding window containing 20 adjacent markers. **(a)** SNP-index (allele frequency) in the D bulk. **(b)** SNP-index in the R bulk. **(c)** Δ(SNP-index), calculated as the absolute value of the difference between the SNP-index of the D bulk and the SNP-index of the R bulk. The shaded area is delimited by the moving averages of the lower and upper bounds of 95% confidence intervals, using the Bonferroni correction for multiple testing (with n=223,711 tests). **(d)** Euclidean distance (dots) and $ED100^4$ (red line). $ED100^4$ values were calculated as described by de la Fuente Cantó et al. (2022). **(e)** G-statistic, calculated as described by Magwene et al. (2011). **(f)** *p*-values of two-tailed Fisher's exact tests. The dashed line marks the Bonferroni-corrected 5% significance threshold, calculated considering that n=223,711 chromosomal locations have been tested.

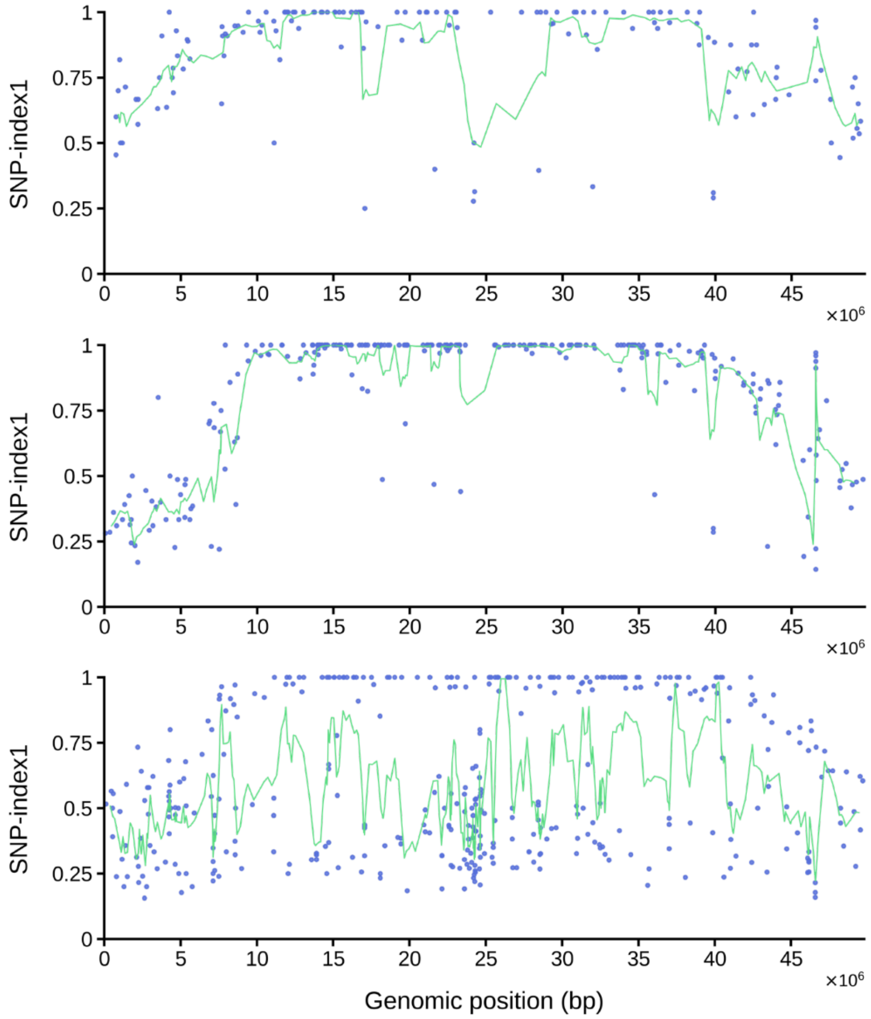

Supplemental Figure 6. Three recessive *eop* mutants of *Arabis alpina* map to chromosome 8. Each dot corresponds to an individual biallelic marker segregating in the population. Unless otherwise stated, continuous lines represent weighted moving averages calculated using a sliding window containing 5 adjacent markers. **(a)** *eop002* mutant. **(b)** *eop085* mutant. **(c)** *eop091* mutant.

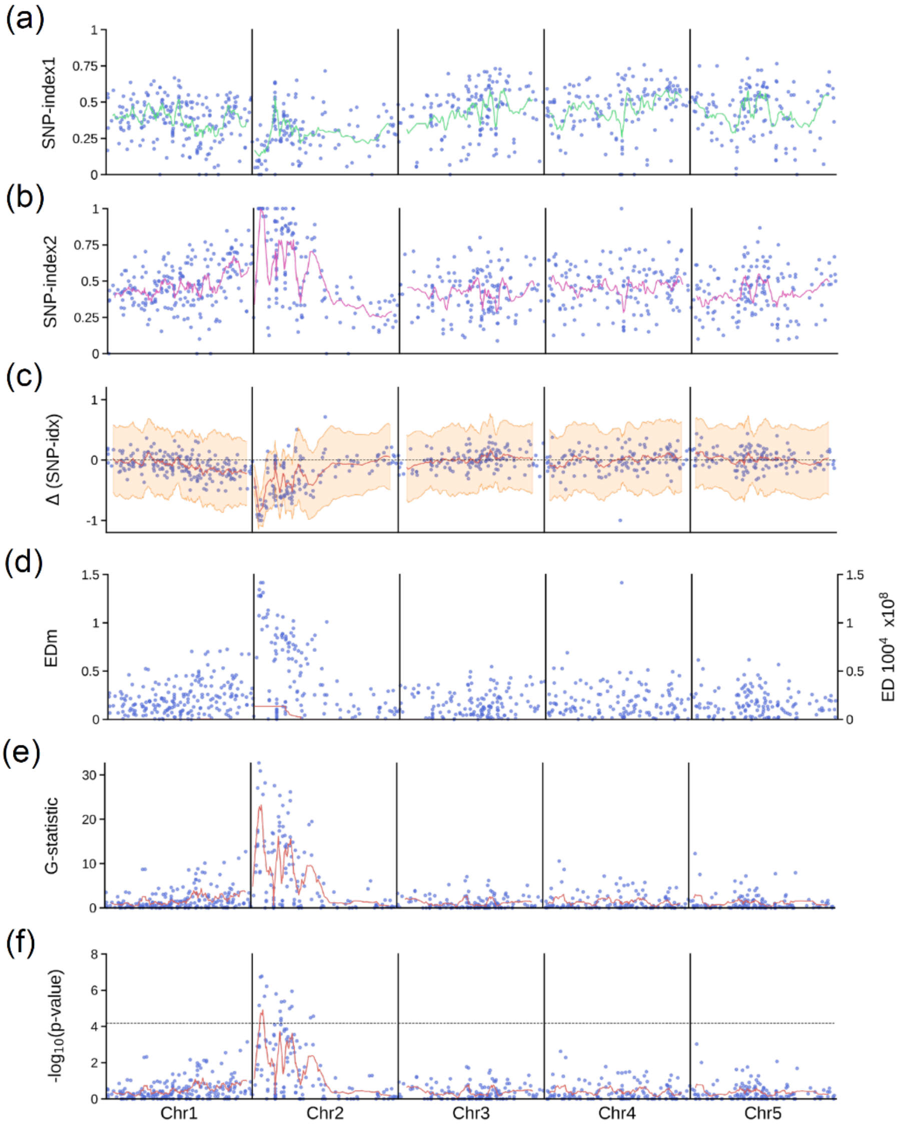

Supplemental Figure 7. Mapping-by-sequencing of an albino mutant of *Arabidopsis thaliana*. Several statistics have been evaluated across all chromosomes, and the results are presented as Manhattan plots. Each dot corresponds to an individual biallelic marker segregating in the population. Continuous lines represent weighted moving averages calculated using a sliding window containing 5 adjacent markers. **(a)** SNP-index (allele frequency) in the D bulk. **(b)** SNP-index in the R bulk. **(c)** Δ(SNP-index), calculated as the difference between the SNP-index of the D bulk and the SNP-index of the D bulk. The shaded area is delimited by the moving averages of the lower and upper bounds of 95% confidence intervals, using the Bonferroni correction for multiple testing (with n=741 tests). **(d)** Euclidean distance (dots) and $ED100^4$ (red line). $ED100^4$ values were calculated as described by de la Fuente Cantó et al. (2022). **(e)** G-statistic, calculated as described by Magwene et al. (2011). **(f)** -log(*p*-value) of two-tailed Fisher's exact tests. The dashed line marks the Bonferroni-corrected 5% significance threshold, calculated considering that n=741 chromosomal locations have been tested.

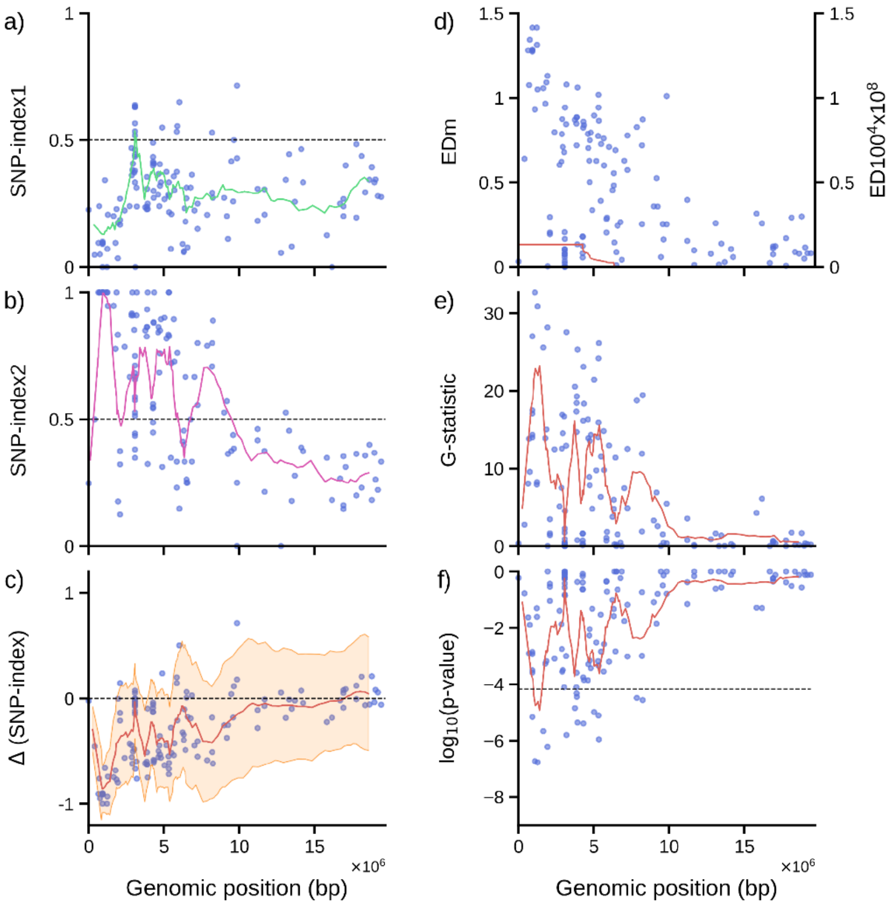

Supplemental Figure 8. Different statistics place an albino mutation on chromosome 2 of *Arabidopsis thaliana*. Each dot corresponds to an individual biallelic marker segregating in the population. Unless otherwise stated, continuous lines represent weighted moving averages calculated using a sliding window containing 5 adjacent markers. **(a)** SNP-index (allele frequency) in the D bulk. **(b)** SNP-index in the R bulk. **(c)** Δ(SNP-index), calculated as the difference between the SNP-index of the D bulk and the SNP-index of the R bulk. The shaded area is delimited by the moving averages of the lower and upper bounds of 95% confidence intervals, using the Bonferroni correction for multiple testing (with n=741 tests). **(d)** Euclidean distance (dots) and $ED100^4$ (red line). $ED100^4$ values were calculated as described by de la Fuente Cantó et al. (2022). **(e)** G-statistic, calculated as described by Magwene et al. (2011). **(f)** *p*-values of two-tailed Fisher's exact tests. The dashed line marks the Bonferroni-corrected 5% significance threshold, calculated considering that n=741 chromosomal locations have been tested.

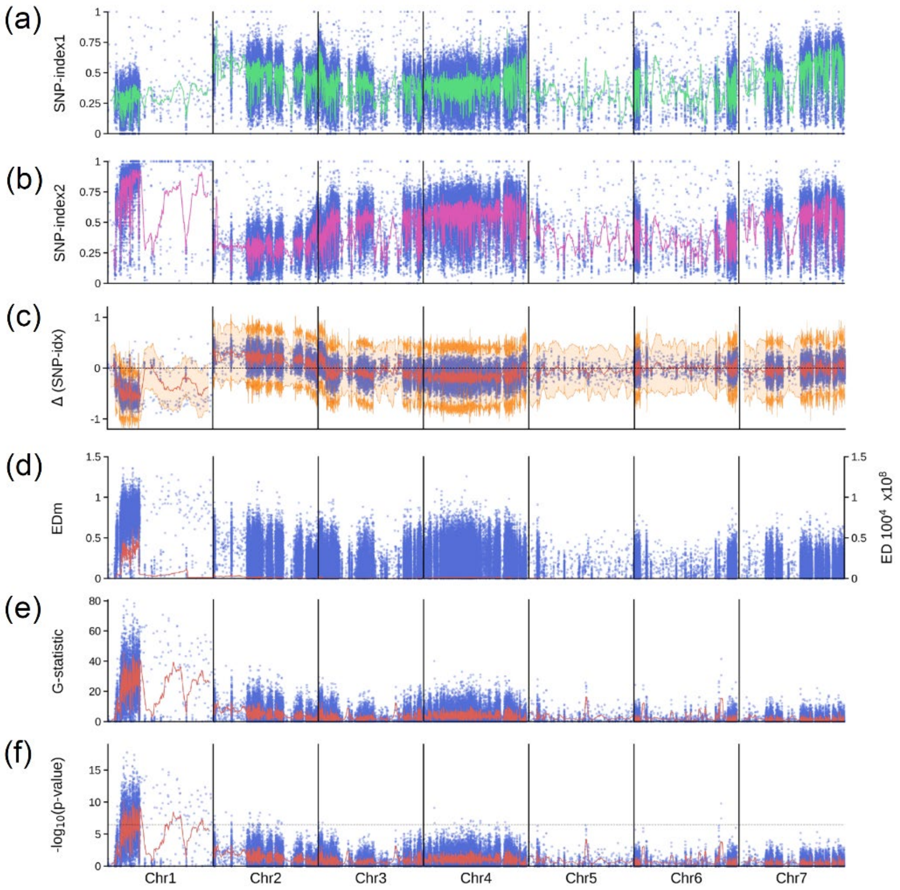

Supplemental Figure 9. Mapping-by-sequencing of the *green petioles-1* mutant of strawberry. Several statistics have been evaluated across all chromosomes, and the results are presented as Manhattan plots. Each dot corresponds to an individual biallelic marker segregating in the population. Unless otherwise stated, continuous lines represent weighted moving averages calculated using a sliding window containing 20 adjacent markers. **(a)** SNP-index (allele frequency) in the D bulk. **(b)** SNP-index in the R bulk. **(c)** Δ(SNP-index), calculated as the difference between the SNP-index of the D bulk and the SNP-index of the D bulk. The shaded area is delimited by the moving averages of the lower and upper bounds of 95% confidence intervals, using the Bonferroni correction for multiple testing (with n=139,639 tests). **(d)** Euclidean distance (dots) and $ED100^4$ (red line). $ED100^4$ values were calculated as described by de la Fuente Cantó et al. (2022). **(e)** G-statistic, calculated as described by Magwene et al. (2011). **(f)** -log(*p*-value) of two-tailed Fisher's exact tests. The dashed line marks the Bonferroni-corrected 5% significance threshold, calculated considering that n=139,639 chromosomal locations have been tested.

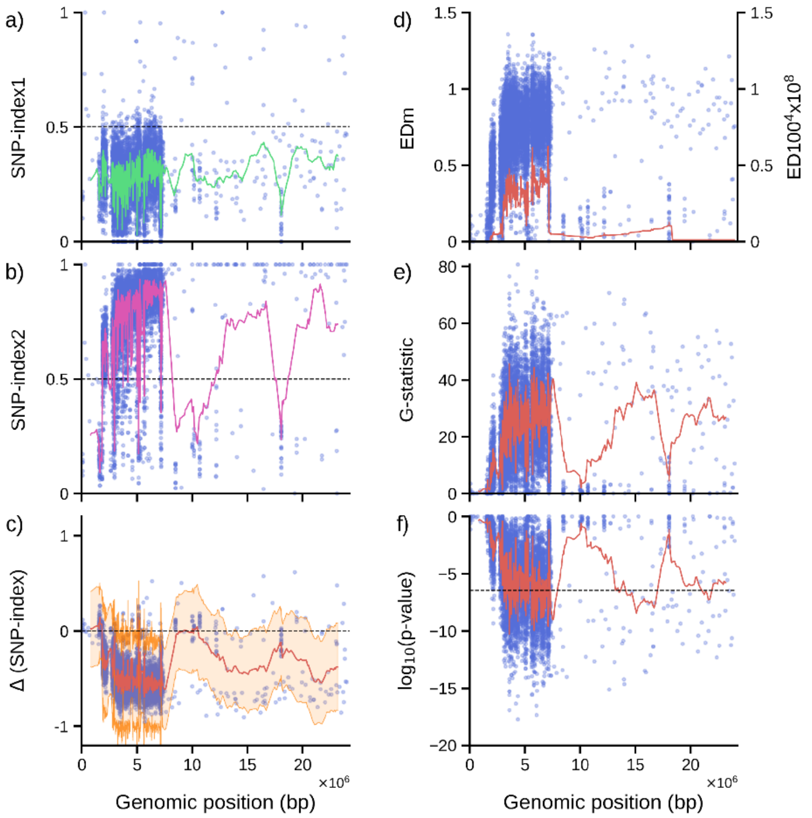

Supplemental Figure 10. Different statistics place the *green petioles-1* mutation on chromosome 1 of strawberry. Each dot corresponds to an individual biallelic marker segregating in the population. Unless otherwise stated, continuous lines represent weighted moving averages calculated using a sliding window containing 20 adjacent markers. **(a)** SNP-index (allele frequency) in the D bulk. **(b)** SNP-index in the R bulk. **(c)** Δ(SNP-index), calculated as the difference between the SNP-index of the D bulk and the SNP-index of the R bulk. The shaded area is delimited by the moving averages of the lower and upper bounds of 95% confidence intervals, using the Bonferroni correction for multiple testing (with n=139,639 tests). **(d)** Euclidean distance (dots) and $ED100^4$ (red line). $ED100^4$ values were calculated as described by de la Fuente Cantó et al. (2022). **(e)** G-statistic, calculated as described by Magwene et al. (2011). **(f)** *p*-values of two-tailed Fisher's exact tests. The dashed line marks the Bonferroni-corrected 5% significance threshold, calculated considering that n=139,639 chromosomal locations have been tested.

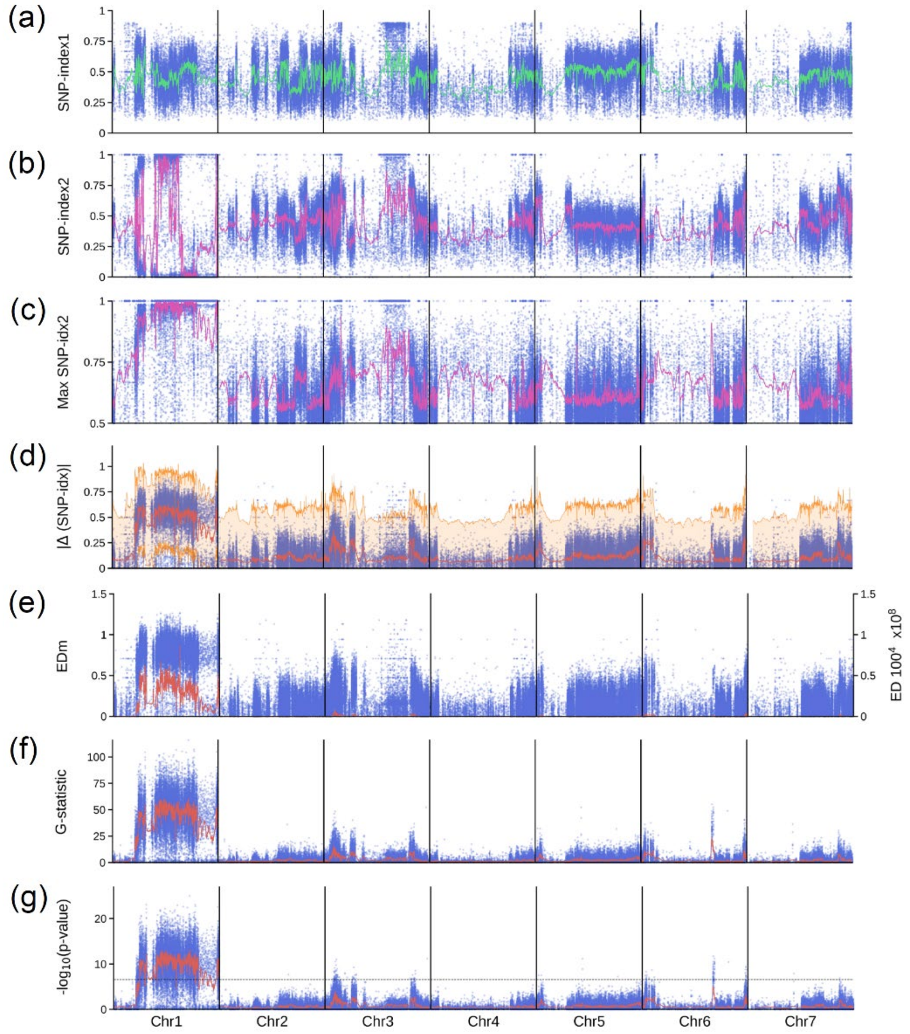

Supplemental Figure 11. Mapping-by-sequencing of a white fruit mutant of strawberry. Several statistics have been evaluated across all chromosomes, and the results are presented as Manhattan plots. Each dot corresponds to an individual biallelic marker segregating in the population. Unless otherwise stated, continuous lines represent weighted moving averages calculated using a sliding window containing 100 adjacent markers. **(a)** SNP-index (allele frequency) in the D bulk. **(b)** SNP-index in the R bulk. **(c)** Allele frequency of the most abundant allele in the R pool. **(d)** |Δ(SNP-index)|, calculated as the absolute value of the difference between the SNP-index of the D bulk and the SNP-index of the R bulk. The shaded area is delimited by the moving averages of the lower and upper bounds of 95% confidence intervals, using the Bonferroni correction for multiple testing (with n=183,155 tests). **(e)** Euclidean distance (dots) and $ED100^4$ (red line). $ED100^4$ values were calculated as described by de la Fuente Cantó et al. (2022). **(f)** G-statistic, calculated as described by Magwene et al. (2011). **(g)** -log(*p*-value) of two-tailed Fisher's exact tests. The dashed line marks the Bonferroni-corrected 5% significance threshold, calculated considering that n=183,155 chromosomal locations have been tested.

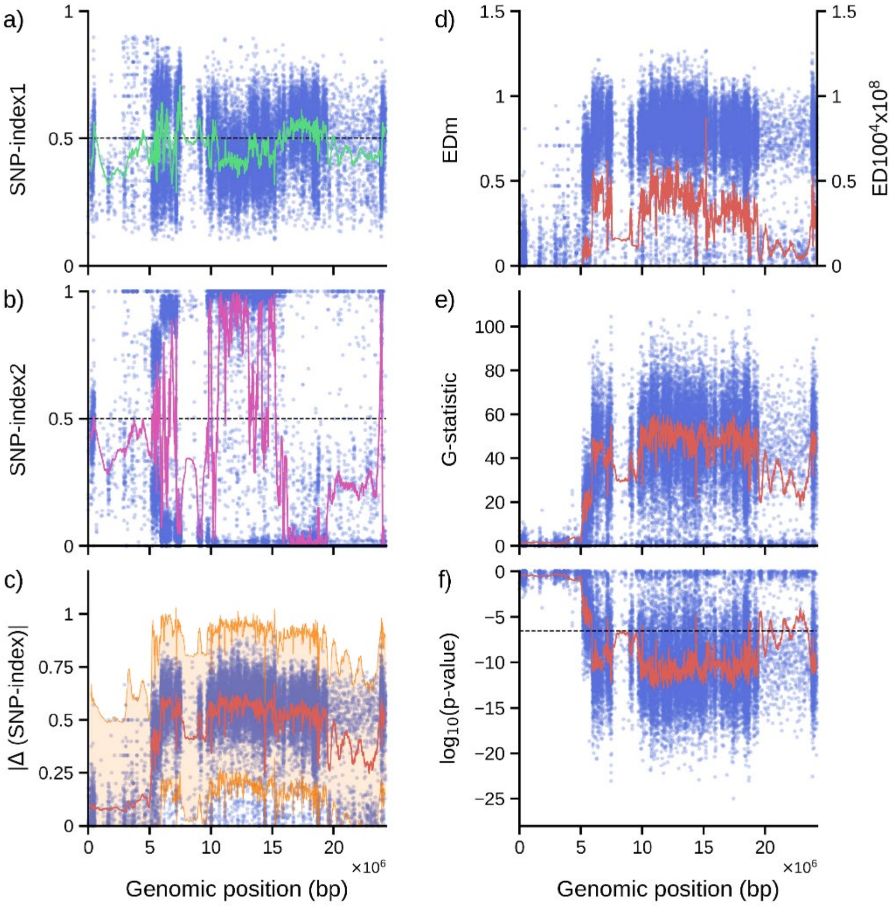

Supplemental Figure 12. Different statistics place a white fruit mutation on chromosome 1 of strawberry. Each dot corresponds to an individual biallelic marker segregating in the population. Unless otherwise stated, continuous lines represent weighted moving averages calculated using a sliding window containing 100 adjacent markers. **(a)** SNP-index (allele frequency) in the D bulk. **(b)** SNP-index in the R bulk. **(c)** |Δ(SNP-index)|, calculated as the absolute value of the difference between the SNP-index of the D bulk and the SNP-index of the R bulk. The shaded area is delimited by the moving averages of the lower and upper bounds of 95% confidence intervals, using the Bonferroni correction for multiple testing (with n=183,155 tests). **(d)** Euclidean distance (dots) and $ED100^4$ (red line). $ED100^4$ values were calculated as described by de la Fuente Cantó et al. (2022). **(e)** G-statistic, calculated as described by Magwene et al. (2011). **(f)** *p*-values of two-tailed Fisher's exact tests. The dashed line marks the Bonferroni-corrected 5% significance threshold, calculated considering that n=183,155 chromosomal locations have been tested.

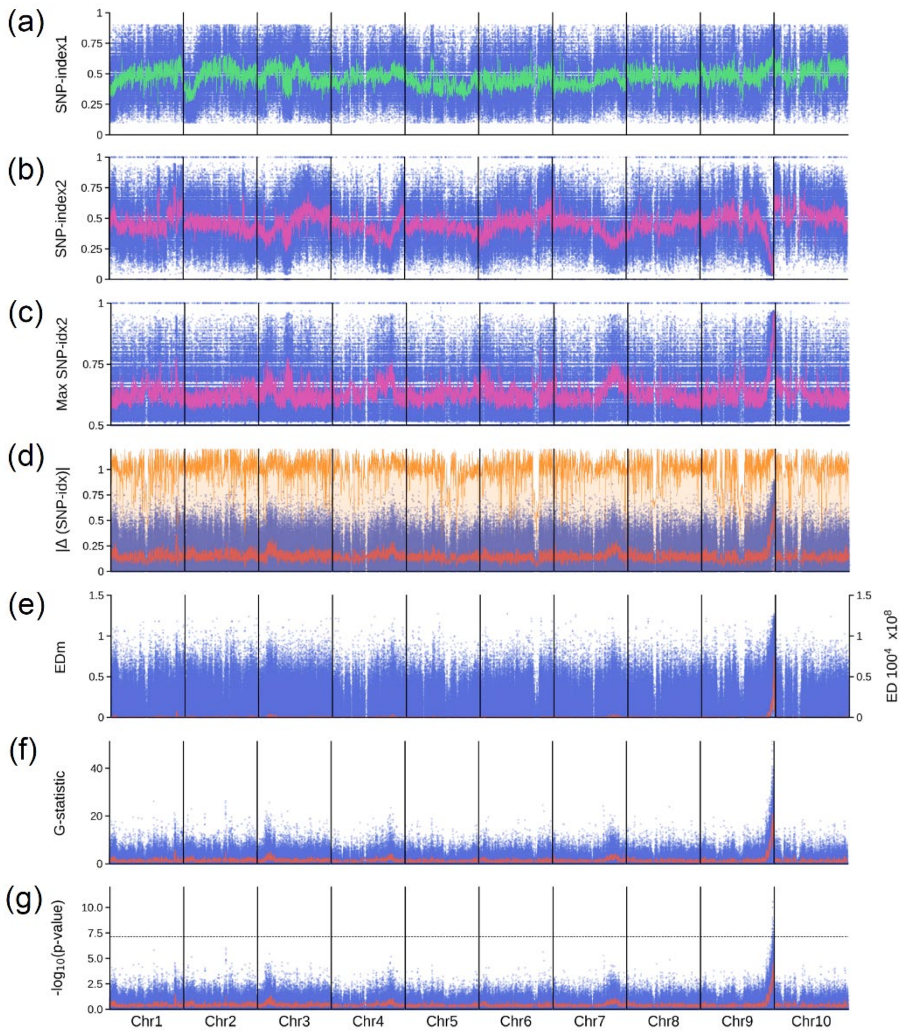

Supplemental Figure 13. Mapping-by-sequencing of a glossy mutant of Chinese cabbage. Several statistics have been evaluated across all chromosomes, and the results are presented as Manhattan plots. Each dot corresponds to an individual biallelic marker segregating in the population. Unless otherwise stated, continuous lines represent weighted moving averages calculated using a sliding window containing 100 adjacent markers. **(a)** SNP-index (allele frequency) in the D bulk. **(b)** SNP-index in the R bulk. **(c)** Allele frequency of the most abundant allele in the R pool. **(d)** |Δ(SNP-index)|, calculated as the absolute value of the difference between the SNP-index of the D bulk and the SNP-index of the R bulk. The shaded area is delimited by the moving averages of the lower and upper bounds of 95% confidence intervals, using the Bonferroni correction for multiple testing (with n=670,109 tests). **(e)** Euclidean distance (dots) and ED100^4 (red line). ED100^4 values were calculated as described by de la Fuente Cantó et al. (2022). **(f)** G-statistic, calculated as described by Magwene et al. (2011). **(g)** -log(*p*-value) of two-tailed Fisher's exact tests. The dashed line marks the Bonferroni-corrected 5% significance threshold, calculated considering that n=670,109 chromosomal locations have been tested.

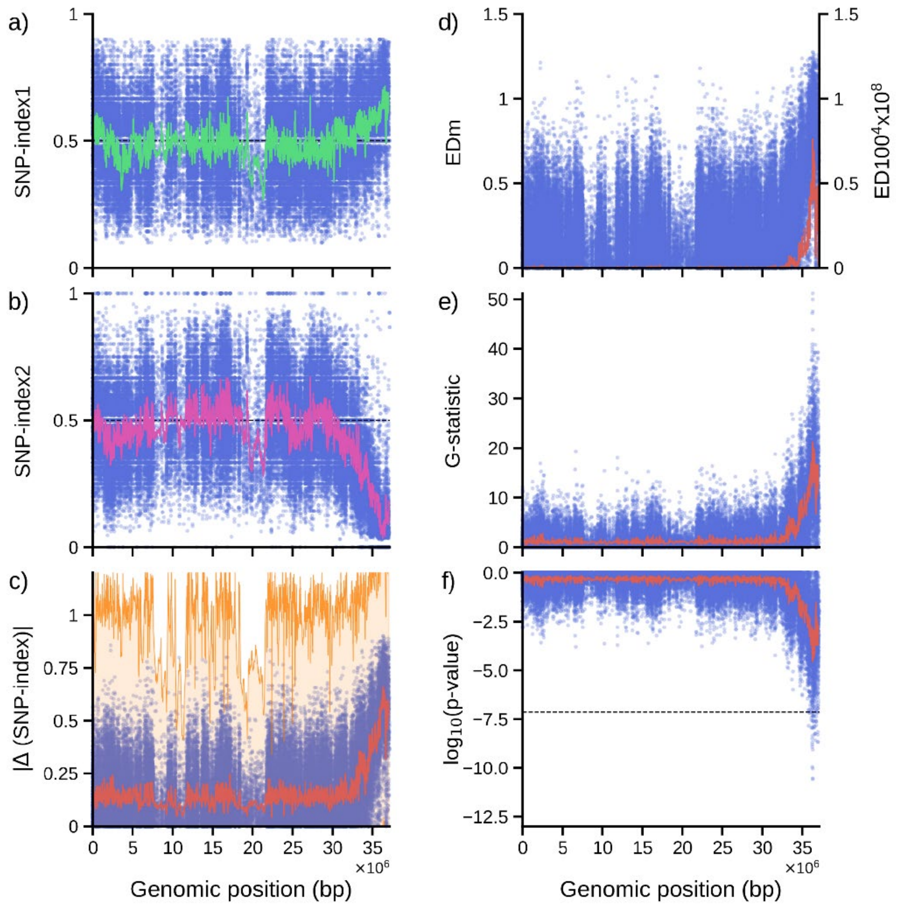

Supplemental Figure 14. Different statistics place a glossy mutation on chromosome 9 of Chinese cabbage. Each dot corresponds to an individual biallelic marker segregating in the population. Unless otherwise stated, continuous lines represent weighted moving averages calculated using a sliding window containing 100 adjacent markers. **(a)** SNP-index (allele frequency) in the D bulk. **(b)** SNP-index in the R bulk. **(c)** |Δ(SNP-index)|, calculated as the absolute value of the difference between the SNP-index of the D bulk and the SNP-index of the R bulk. The shaded area is delimited by the moving averages of the lower and upper bounds of 95% confidence intervals, using the Bonferroni correction for multiple testing (with n=670,109 tests). **(d)** Euclidean distance (dots) and $ED100^4$ (red line). $ED100^4$ values were calculated as described by de la Fuente Cantó et al. (2022). **(e)** G-statistic, calculated as described by Magwene et al. (2011). **(f)** *p*-values of two-tailed Fisher's exact tests. The dashed line marks the Bonferroni-corrected 5% significance threshold, calculated considering that n=670,109 chromosomal locations have been tested.

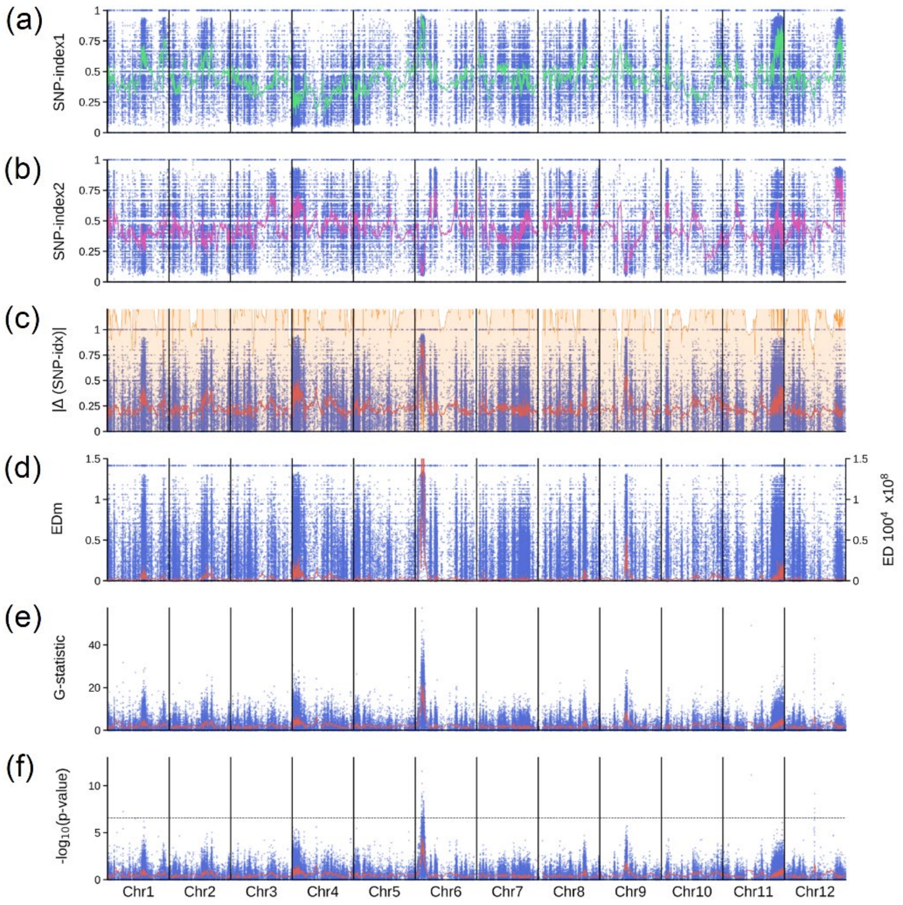

Supplemental Figure 15. QTL-seq of blast disease resistance in rice. Several statistics have been evaluated along all chromosomes, and the results are presented as Manhattan plots. Each dot corresponds to an individual biallelic marker segregating in the population. Unless otherwise stated, continuous lines represent weighted moving averages calculated using a sliding window containing 100 adjacent markers. **(a)** SNP-index (allele frequency) in the H bulk. **(b)** SNP-index in the L bulk. **(c)** |Δ(SNP-index)|, calculated as the absolute value of the difference between the SNP-index of the H bulk and the SNP-index of the L bulk. The shaded area is delimited by the moving averages of the lower and upper bounds of 95% confidence intervals, using the Bonferroni correction for multiple testing (with n=187,181 tests). **(d)** Euclidean distance (dots) and $ED100^4$ (red line). $ED100^4$ values were calculated as described by de la Fuente Cantó et al. (2022). **(e)** G-statistic, calculated as described by Magwene et al. (2011). **(f)** -log(*p*-value) of two-tailed Fisher's exact tests. The dashed line marks the Bonferroni-corrected 5% significance threshold, calculated considering that n=187,181 chromosomal locations have been tested.

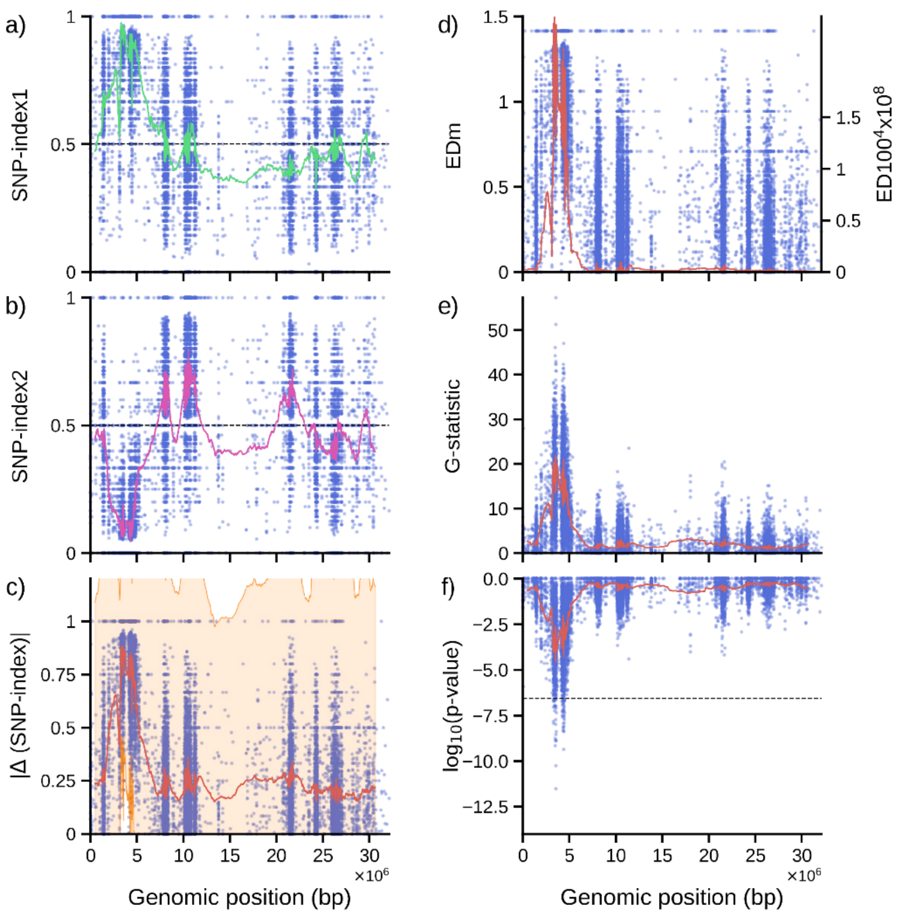

Supplemental Figure 16. Different statistics place a QTL for blast disease resistance on chromosome 6 of rice. Each dot corresponds to an individual biallelic marker segregating in the population. Unless otherwise stated, continuous lines represent weighted moving averages calculated using a sliding window containing 100 adjacent markers. **(a)** SNP-index (allele frequency) in the H bulk. **(b)** SNP-index in the L bulk. **(c)** |Δ(SNP-index)|, calculated as the absolute value of the difference between the SNP-index of the H bulk and the SNP-index of the L bulk. The shaded area is delimited by the moving averages of the lower and upper bounds of 95% confidence intervals, using the Bonferroni correction for multiple testing (with n=187,181 tests). **(d)** Euclidean distance (dots) and $ED100^4$ (red line). $ED100^4$ values were calculated as described by de la Fuente Cantó et al. (2022). **(e)** G-statistic, calculated as described by Magwene et al. (2011). **(f)** *p*-values of two-tailed Fisher's exact tests. The dashed line marks the Bonferroni-corrected 5% significance threshold, calculated considering that n=187,181 chromosomal locations have been tested.

Supplemental Table 1. Options used with the `mbs` command in each case study.

Case	Options	Reference
1	-d D,R,Pr,Pd -m R --parental filter -I --EMS	Cao et al. 2019
2	-d D,R,Pd,Pr -m R -c 8 --parental-filter	Jiang et al.
3	-d D,R,Pd -m R -c 8 --parental-filter -I --EMS	Bournonville et al. 2023
4	-d R,Pd,Pr -m R --parental filter -I --EMS	Huang et al. 2022
5	-d R,Pd -m R --parental-filter --EMS	Viñegra de la Torre et al. 2022
6	-d D,R,Wr -m R -r D --parental-filter --EMS	Rodríguez-Alcocer et al. 2023
7	-d R,D -m R -r D -c 8 -I	Luo et al. 2023
8	-d R,D -m R --het-filter -q 10 -Q 90 -I	Castillejo et al. 2020
9	-d D,R -m R --het-filter -q 10 -Q 90 -I	Yang et al. 2022
10	-d H,L	Takagi et al 2013
